# Molecular phylogeny of the subfamily Stevardiinae Gill, 1858 (Characiformes: Characidae): classification and the evolution of reproductive traits

**DOI:** 10.1186/s12862-015-0403-4

**Published:** 2015-07-21

**Authors:** Andréa T. Thomaz, Dahiana Arcila, Guillermo Ortí, Luiz R. Malabarba

**Affiliations:** Department of Ecology and Evolutionary Biology (EEB), University of Michigan, 1109 Geddes Ave., Ann Arbor, 48109 MI USA; Departamento de Zoologia, Universidade Federal do Rio Grande do Sul (UFRGS), Av. Bento Gonçalves 9500, Porto Alegre, 90501-970 RS Brazil; Department of Biological Sciences, The George Washington University, 2023 G St. NW, Washington, DC 20052 USA; Department of Vertebrate Zoology, National Museum of Natural History Smithsonian Institution, PO Box 37012, MRC 159, Washington, DC 20013 USA

**Keywords:** Tetras, Neotropical region, “Clade A”, Stevardiinae, Glandulocaudinae, Insemination, Multi-locus phylogeny

## Abstract

**Background:**

The subfamily Stevardiinae is a diverse and widely distributed clade of freshwater fishes from South and Central America, commonly known as “tetras” (Characidae). The group was named “clade A” when first proposed as a monophyletic unit of Characidae and later designated as a subfamily. Stevardiinae includes 48 genera and around 310 valid species with many species presenting inseminating reproductive strategy. No global hypothesis of relationships is available for this group and currently many genera are listed as *incertae sedis* or are suspected to be non-monophyletic.

**Results:**

We present a molecular phylogeny with the largest number of stevardiine species analyzed so far, including 355 samples representing 153 putative species distributed in 32 genera, to test the group’s monophyly and internal relationships. The phylogeny was inferred using DNA sequence data from seven gene fragments (mtDNA: *12S*, *16S* and *COI*; nuclear: *RAG1*, *RAG2*, *MYH6* and *PTR*). The results support the Stevardiinae as a monophyletic group and a detailed hypothesis of the internal relationships for this subfamily.

**Conclusions:**

A revised classification based on the molecular phylogeny is proposed that includes seven tribes and also defines monophyletic genera, including a resurrected genus *Eretmobrycon*, and new definitions for *Diapoma*, *Hemibrycon*, *Bryconamericus sensu stricto*, and *Knodus sensu stricto*, placing some small genera as junior synonyms. Inseminating species are distributed in several clades suggesting that reproductive strategy is evolutionarily labile in this group of fishes.

**Electronic supplementary material:**

The online version of this article (doi:10.1186/s12862-015-0403-4) contains supplementary material, which is available to authorized users.

## Background

The family Characidae is the largest family of freshwater fishes in the Neotropics, comprising around 1065 species in approximately 146 genera [1]. Because of its considerable species richness and diversity, the relationships and limits of the main lineages in the family have been controversial: two-thirds of all species were considered *incertae sedis* in the family just a decade ago due to lack of consistent information on their relationships [2]. Besides species richness, a primary challenge to establishing relationships based on morphological phylogenies has been associated with their conservative morphology though their long period of evolution, since characid fossils with essentially modern morphologies are known from Eocene-Oligocene deposits [3]. Taken together, these factors may explain the high level of morphological homoplasy inferred across lineages within the family (e.g. [4, 5]). In recent years, an increasing understanding of the relationships among major lineages within Characidae is emerging on the basis of evidence provided by molecules, osteology, and primary and secondary sexual characters [4, 6–12].

**Fig. 1:**
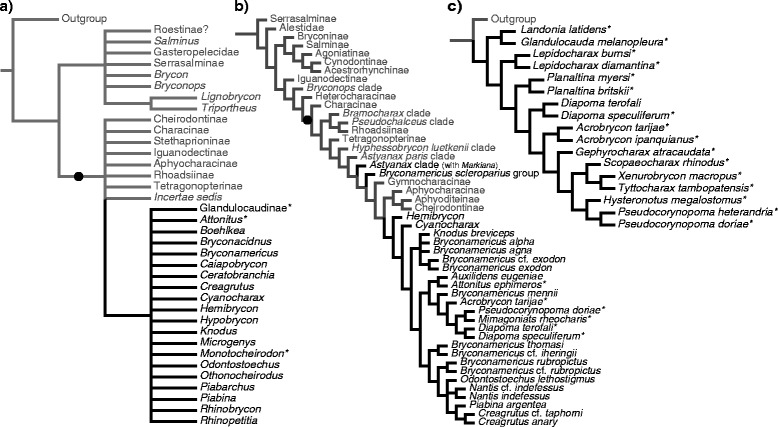
Relationships of Stevardiinae based on morphological studies. Phylogenetic relationships among major groups of characid and stevardiin taxa according to morphological analyses by (**a**) Malabarba and Weitzman [6], (**b**) Mirande [4], and (**c**) Ferreira et al. [20]. Black branches and names indicate Stevardiinae taxa. The black circle on the internal branches indicates the synapomorphic loss of the supraorbital bone in (**a**) and (**b**), a diagnostic character for Characidae *sensu* Oliveira et al. [12] (except for Iguanodectinae in A). Asterisks (*) indicate taxa with inseminating strategy

**Fig. 2:**
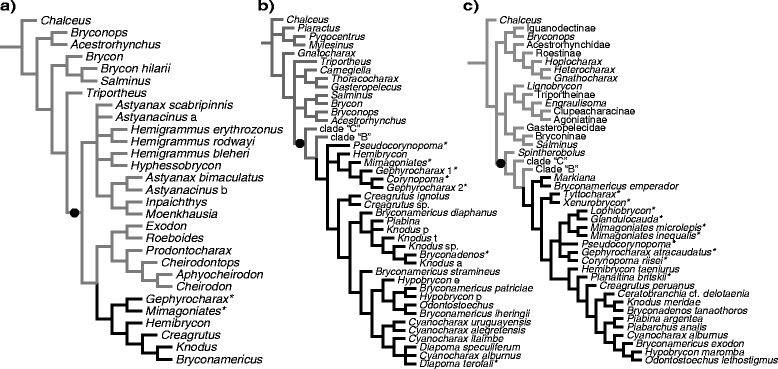
Relationships of Stevardiinae based on previous molecular studies. Phylogenetic relationships among major groups of characid and stevardiin fishes according to molecular analyses by (**a**) Calcagnotto et al. [7], (**b**) Javonillo et al. [10] and (**c**) Oliveira et al. [12]. Black branches and names indicate Stevardiinae taxa. The black circle on the internal branches indicates the synapomorphic loss of the supraorbital bone in (**a**) and (**b**), a diagnostic character for Characidae *sensu* Oliveira et al. [12]. Asterisks (*) indicate taxa with inseminating strategy

Two large and subordinated clades of interest in our investigation have been successively recognized as monophyletic in Characidae. The first more inclusive clade embraces all characid species that lack a supraorbital bone [4, 6–8, 10, 12] (Figs. 1 and 2). This clade was recently raised to family rank and named Characidae in a more restricted sense by previous authors [12], who also reassigned all characid species bearing a supraorbital bone to new or previously recognized characiform families. The second, less inclusive clade and the main subject of this study, was informally named “clade A” and diagnosed based on two synapomorphies: the dorsal fin with two unbranched and eight branched rays, and the premaxilla with four teeth in the inner series [6]. “Clade A”, *sensu* Malabarba and Weitzman [6], included the subfamily Glandulocaudinae Eigenmann, 1914 *sensu* Weitzman and Menezes [13] (with 19 genera), the newly described genus *Cyanocharax,* and 18 genera of uncertain relationships previously listed within Cheirodontinae or Tetragonopterinae [14] and classified as *incertae sedis* genera in Characidae [2]: *Attonitus, Boehlkea, Bryconacidnus, Bryconamericus, Caiapobrycon, Ceratobranchia, Creagrutus, Hemibrycon, Hypobrycon, Knodus, Microgenys, Monotocheirodon, Odontostoechus, Othonocheirodus*, *Piabarchus, Piabina, Rhinobrycon,* and *Rhinopetitia* (Fig. 1a, Table 1). Prior to this proposition [6], nothing was known about phylogenetic relationships among these 18 *incertae sedis* genera, except for two studies involving *Caiapobrycon*, *Creagrutus* and *Piabina* [15, 16] . Conversely, phylogenies were available at the tribe and genus levels for all taxa previously included in the Glandulocaudinae, mostly based on sexually dimorphic characters of males [13].

**Table 1:** Current classification of Stevardiinae

Tribe	Genera	No. of species
**Diapomini**	***Diapoma*** Cope, 1894	4
	*Planaltina* Böhlke, 1954	3
	***Acrobrycon*** Eigenmann and Pearson, 1924	2
**Glandulocaudini**	***Glandulocauda*** Eigenmann, 1911	2
	***Lophiobrycon*** Castro, Ribeiro, Benine and Melo, 2003 [36]	1
	***Mimagoniates*** Regan, 1907	7
**Hysteronotini**	*Hysteronotus* Eigenmann, 1911	1
	***Pseudocorynopoma*** Perugia, 1891	2
**Landonini**	***Landonia*** Eigenmann and Henn, 1914	1
**Stevardiini**	***Corynopoma*** Gill, 1858	1
	***Gephyrocharax*** Eigenmann, 1912	13
	*Pterobrycon* Eigenmann, 1913	2
**Phenacobryconini**	*Phenacobrycon* Eigenmann, 1922	1
**Xenurobryconini**	***Argopleura*** Eigenmann, 1913	4
	***Chrysobrycon*** Weitzman and Menezes, 1998	3
	*Iotabrycon* Roberts, 1973	1
	*Ptychocharax* Weitzman, Fink, Machado-Allison and Royero, 1994	1
	***Scopaeocharax*** Weitzman and Fink, 1985	2
	***Tyttocharax*** Fowler, 1913	6
	***Xenurobrycon*** Myers and Miranda Ribeiro, 1945	5
***Incertae Sedis***	***Attonitus*** Vari and Ortega, 2000	3
	*Aulixidens* Böhlke, 1952 [8]	1
	*Boehlkea* Géry, 1966	2
	***Bryconacidnus*** Myers, 1929	3
	***Bryconadenos*** Weitzman, Menezes, Evers and Burns, 2005 [19]	2
	***Bryconamericus*** Eigenmann, 1907	78
	*Caiapobrycon* Malabarba and Vari, 2000	1
	***Carlastyanax*** Géry, 1972 [21]	1
	***Ceratobranchia*** Eigenmann, 1914	5
	***Creagrutus*** Günther, 1864	71
	***Cyanocharax*** Malabarba and Weitzman, 2003	8
	*Cyanogaster* Mattox, Britz, Toledo-Piza and Marinho, 2013 [66]	1
	***Hemibrycon*** Günther, 1864	31
	***Hypobrycon*** Malabarba and Malabarba, 1994	3
	***Knodus*** Eigenmann, 1911	20
	***Lepidocharax*** Ferreira, Menezes and Quagio-Grassiotto, 2011 [20]	2
	***Markiana*** Eigenmann, 1903 [31]	2
	*Microgenys* Eigenmann, 1913	3
	*Monotocheirodon* Eigenmann and Pearson, 1924	1
	***Nantis*** Mirande, Aguilera and Azpelicueta, 2006 [8]	1
	***Odontostoechus*** Gomes, 1947	1
	*Othonocheirodus* Myers, 1927	1
	*Phallobrycon* Menezes, Ferreira and Netto-Ferreira, 2009 [67]	1
	***Piabarchus*** Myers, 1928	2
	***Piabina*** Reinhardt, 1867	2
	***Rhinobrycon*** Myers, 1944	1
	*Rhinopetitia* Géry, 1964	1
	*Trochilocharax* Zarske, 2010 [68]	1

Tribes and genera currently recognized in Stevardiinae (“clade A”). Composition of the tribes Diapomini, Glandulocaudini, Hysteronotini, Landonini, Phenacobryconini, Stevardiini, and Xenurobryconini follows [13, 19, 36]. Genera originally included in Stevardiinae [6] or subsequently by other authors indicated by superscripts matching the reference number [8, 19–21, 31, 36, 66–68]; Genera in bold were included in this study

The presence of modified scales in the caudal fin of males has been the main character defining the Glandulocaudinae since the group was described [17]. The complex morphologies of these caudal organs were explored in detail in phylogenetic studies to test the monophyly and internal relationships of the glandulocaudin tribe Xenurobryconini [18] and to diagnose and propose internal relationships within Glandulocaudinae. On this basis, Glandulocaudinae was divided into seven tribes [13]: Corynopomini Eigenmann, 1927 (renamed Stevardiini in 2005 [19] – see below), Diapomini Eigenmann, 1910, Glandulocaudini Eigenmann, 1914, Hysteronotini Eigenmann, 1914, Landonini Weitzman and Menezes, 1998, Phenacobryconini Weitzman and Menezes, 1998 and Xenurobryconini Myers and Böhlke, 1956 (Table 1). This classification was further modified in light of new histological evidence from the caudal organs of males [19]. As a consequence, the subfamily Glandulocaudinae was restricted to the tribe Glandulocaudini *sensu* Weitzman and Menezes [13] and the six remaining tribes were placed in the resurrected subfamily Stevardiinae [19]. The family group name Corynopomini is a junior synonym, being this tribe consequently also renamed as Stevardiini (see Additional file 1 for nomenclatural remarks).

Monophyly of “clade A”, including the Glandulocaudinae and Stevardiinae *sensu* Weitzman, Menezes, Evers and Burns [19] and the 18 *incertae sedis* genera listed previously [6], has been supported more recently on the basis of both morphological [4, 11, 20, 21] (Fig. 1b, c) and molecular data [7, 10, 12] (Fig. 2). Furthermore, later studies have added ten genera to “clade A” after the original definition [6], such that it currently includes 48 genera (Table 1). In a morphological phylogeny of 160 characiform species, including 23 species and 14 genera of “clade A”, the Stevardiinae *sensu* Weitzman and collaborators [19] was reported to be paraphyletic given that it also included Glandulocaudinae [4, 8] (Fig. 1b). Based on this inference, a more comprehensive concept of the name Stevardiinae was advanced embracing all members of “clade A”, with Glandulocaudinae being lowered in rank to a monophyletic tribe (Glandulocaudini) within Stevardiinae [8] (Table 1, see nomenclatural remarks in Additional file 1). The newly defined Stevardiinae *sensu* Mirande [8] was diagnosed on the basis of three synapomorphies: (i) the previously mentioned possession of eight branched dorsal-fin rays [6], (ii) the absence of the epiphyseal branch of the supraorbital canal, and (iii) the presence of nine dorsal-fin pterygiophores. For clarity, “clade A” was then named Stevardiinae and, as presently recognized, is widely distributed in the Neotropics on both sides of the Andes, from Costa Rica in Central America to central Argentina.

Several species in Stevardiinae have been shown to be inseminating [22] (Table 2), a term used to describe the capacity of males to transfer sperm directly into the female reproductive tract. Possible selective advantages of insemination include increase of the probability of fertilization, protection of the gametes from a potentially harmful environment, the temporal and spatial separation of mating and oviposition, and sperm competition [23]. Also, this strategy in characids is correlated with small-sized species that produce fewer numbers of eggs (<500 oocytes) [9]. In Stevardiinae, inseminating species tend to have lower relative fecundity values than other characids and some species are able to reproduce during major part of the year (e.g., *Mimagoniates* and *Pseudocorynopoma*), which may demonstrate the advantages of the insemination strategy [9].

**Table 2:** Insemination strategy and sperm morphology

Tribes Genera	Inseminating species	Non-inseminating species^a^	Sperm morph.	Species analyzed for sperm morphology
**Diapomini**				
*Acrobrycon*	*A. ipanquianus* [22]*,* *Acrobrycon* sp. [69]			
*Diapoma*	*D. speculiferum* [69]*, D. terofali* [69]		M2	*D. speculiferum* [70]
*Planaltina*	*P. britiskii* [22]*, P. glandipedis* [47]*,* *P. myersi* [69]			
**Glandulocaudini**				
*Glandulocauda*	*G. melanogenys* [69]*,* *G. melanopleura* [23]			
*Lophiobrycon*	*L. weitzmani* [36]			
*Mimagoniates*	*M. barberi* [69]*, M. inequalis* [22]*,* *M. lateralis* [69]*, M. microlepis* [69]*,* *M. rheocharis* [69]*, M. sylvicola* [69]		M2	*M. barberi* [71]*,* *M. microlepis* [70]
**Hysteronotini**				
*Hysteronotus*	*H. megalostomus* [69]			
*Pseudocorynopoma*	*P. doriae* [69]*,* *P. heterandria* [69]		M2	*P. doriae* [70]
**Landonini**				
*Landonia*	*L. latidens* [69]			
**Phenacobryconini**				
*Phenacobrycon*	*P. henna* [69]			
**Stevardiini**				
*Corynopoma*	*C. riise* [69]		M2	*C. riisei* [72]
*Gephyrocharax*	*G. atracaudatus* [69]*, G. chocoensis* [69]*, G. intermedius* [69]*, G. melanocheir* [22]*, G. valencia* [69]*, G. venezuelae* [69]*,* *Gephyrocharax* sp*.* [34]		M2	*G. atracaudata* [73]*,* *G. intermedius* [73]
*Pterobrycon*	*P. landoni* [22]*, P. myrnae* [69]			
**Xenurobryconini**				
*Argopleura*	*A. chocoensis* [69]*,* *A. magdalensis* [69]			
*Chrysobrycon*	*C. hesperus* [69]*, C. myersi* [69]		M2	*Chrysobrycon* sp*.* [73]
*Iotabrycon*	*I. praecox* [69]			
*Ptychocharax*	*P. rhyacophila* [69]			
*Scopaeocharax*	*S. rhinodus* [69]*,* *Scopaeocharax* sp*.* [69]		M3	*S. rhinodus* [74]
*Tyttocharax*	*T. tambopatensis* [22]*,* *Tyttocharax* sp. [69]		M3	*T. cochui* [74], *T. tambopatensis* [74]
*Xenurobrycon*	*X. macropus* [23]*,* *X. polyancistrus* [69]		M3	*X. heterodon* [75]*,* *X. macropus* [75], *X. polyancistrus* [75]
***Incertae sedis***				
*Attonitus*	*Attonitus bounites* [22]*,* *A. ephimeros* [76]*, A. irisae* [22]			
*Boehlkea*		*B. fredcochui*	M1	*B. fredcochui* [31]
*Bryconadenos*	*B. tanaothoros* [76]		M2	*B. tanaothoros* [19]
*Bryconacidnus*			M1	*B. ellisi* [31]
*Bryconamericus*	*B. pectinatus* [76]	*B. alpha,* *B. deuterodonoides,* *B. exodon, B. iheringii,* *B. pachacuti, Bryconamericus* sp*.*	M1	*B. exodon* [31]
*Caiapobrycon*		*C. tucurui*		
*Ceratobranchia*		*C. binghami*	M1	*C. obtusirostris* [31]
*Creagrutus*	*C. lepidus* [22]*,* *C. melasma* [22]	*C. affinis, C. britskii,* *C. changae, C. cochui, C. figueiredoi, C. holmi, C. menezesi,* *C. paralacus,* *C. taphorni* *Creagrutus* sp.	M1	*C. meridionalis* [31]
*Cyanocharax*		*C. alburnus,* *C. alegretensis,* *C. dicropotamicus,* *C. itaimbe,* *C. lepiclastus,* *C. macropinna*	M1	*C. alburnus* [31]
*Hemibrycon*		*H. dariensis, H. metae*		
*Hypobrycon*		*Hypobrycon* sp*.*		
*Knodus*	*Knodus* sp*.* [69]	*K. beta, K. breviceps,* *K. meridae,* *K. septentrionalis,* *K. turiuba, Knodus* sp*.*	M1	*K. meridae* [31]
*Markiana*		*M. nigripinnis*		
*Monotocheirodon*	*M. pearsoni* [23]*,* *Monotocheirodon* sp*.* [69, 76]			
*Odontostoechus*			M1	*O. lethostigmus* [31]
*Piabarchus*		*P. analis*		
*Piabina*		*P. argentea*	M1	*P. anhembi* [31]*,* *P. argentea* [31]
*Rhinobrycon*		*R. negrensis*	M1	*R. negrensis* [31]

Stevardiinae species with known insemination strategy and sperm morphology, based on several studies indicated by superscripts matching the reference number [19, 22, 23, 31, 34, 36, 47, 69–76]. Taxa not listed in this table indicate lack of information

^a^Indicates personal communication by John Burns

Except for two species of *Monotocheirodon* that bear intromittent organs, all remaining species of Stevardiinae lack copulatory organs [24, 25]. Among these, insemination strategy has been documented by the presence of sperm in females through histological examination of the ovaries for all species of the tribes Diapomini, Glandulocaudini, Hysteronotini, Landonini, Phenacobryconini, Stevardiini and Xenurobryconini, all the species of *Attonitus*, *Bryconadenos* and *Monotocheirodon* and some species of *Bryconamericus* (*B. pectinatus*), *Creagrutus* (*C. lepidus* and *C. melasma*) and *Knodus* (*Knodus* sp.) [23] (Table 2). Inseminating species, however, also have been described in other lineages of Characidae, such as the tribe Compsurini within the subfamily Cheirodontinae [26] and in a clade formed by *Hollandichthys* plus *Rachoviscus* [27]. Taking this pattern at face value, insemination seems to have at least three independent origins within Characidae [28].

The presence of insemination correlates with differences in sperm morphology. While in most externally fertilizing teleosts the spermatozoa are characterized by a spherical to ovoid nucleus and short midpiece, in inseminating fishes an elongated nucleus is the most frequently observed [23, 27]. This elongation may be advantageous for insemination over ovoid nucleus since it facilitates sperm movement through the female gonopore and within the female reproductive tract, increasing directional movement toward female gonopore, and facilitating the formation of sperm packets to be moved by the male urogenital papilla to the female urogenital pore [23].

Three unique morphotypes of sperm have been described among species of Stevardiinae (M1, M2, and M3; Table 2), based on arrangement of centrioles, flagellum, nucleus, and the midpiece [11] (Table 2). Based on the phylogenetic evidence available [19], it has been proposed that insemination is likely to have evolved only once within Stevardiinae and may constitute a synapomorphy shared by the most derived species of this subfamily [11]. However, recent morphological phylogenies [4, 8, 20, 21] (Fig. 1) still have limited taxonomic representation to effectively test this hypothesis which is, likewise, not supported by molecular evidence given the absence of a monophyletic group with all inseminating stevardiines [10, 12] (Fig. 2). In terms of sperm morphology, it has been proposed that sperm type M1 is synapomorphic to Stevardiinae, M2 synapomorphic to all the inseminating species of the Stevardiinae, and M3 synapomorphic to the Xenurobryconini [11]. Information about these traits related to reproduction, however, are just known for less than 1/3 of all stevardiines species, which hamper a better understading of the evolution of the reproductive strategy in this subfamily.

Most of the species-level diversity in Stevardiinae is contained in only four genera (out of 48) that include 200 out of 311 nominal species described for the subfamily (Table 1). Among these four genera, *Creagrutus* (71 species) is the only one supported as a monophyletic group based on apomorphic features associated to jaws and teeth [16]. The remaining three genera *Bryconamericus* (78 species), *Hemibrycon* (31 species) and *Knodus* (20 species) have been traditionally and arbitrarily diagnosed using pre-cladistic criteria based on the number of teeth on the maxilla and on the extension of scales over the caudal-fin rays [29]. Not surprisingly, recently published morphological- and molecular-based characid phylogenies found that *Bryconamericus* and *Knodus* are polyphyletic groups (Fig. 1b [4]; Fig. 2b–c [10, 12]), thus demonstrating the need of further study to diagnose monophyletic genera based on consistent phylogenetic evidence.

This study presents phylogenetic relationships for a large number of taxa of Stevardiinae based on analysis of a multi-locus data set to address two main goals: (i) test the monophyly of the Stevardiinae and the included putative tribes and genera, with emphasis on the species-rich genera *Bryconamericus, Hemibrycon,* and *Knodus*; and (ii) shed light on the evolution of insemination and secondary sexual dimorphism among stevardiines, specifically whether insemination had a single origin among members of this subfamily.

## Results

Sequences from three mitochondrial (ribosomal *12S* and *16S* rRNA subunits, and cytochrome oxidase I - *COI*) and four nuclear loci (exon regions of myosin, heavy polypeptide 6 - *MYH6*; hypothetical protein LOC564097 – *PTR*; recombination activating gene 1 - *RAG1*; and gene 2 - *RAG2*) were obtained from a total of 355 individuals. The concatenated alignment contains 4,816 sites, of which 1,920 are variable. Some markers could not be successfully amplified and sequenced for a number of taxa due to technical issues or low quality of the genomic DNA. Mitochondrial and nuclear DNA sequences could be obtained for 85 % and 69 % of the taxa, respectively. Efficiency for nuclear genes was lower, most likely due to non-conserved priming regions and a higher risk of cross-contamination in the nested PCR procedure (some sequences were eliminated after contamination was diagnosed by our quality-control protocol). Overall, the data set is 76 % complete. More detailed information for each molecular marker can be found in Table 3 and for sequences obtained for each specimen in Additional file 2. The best-fit partitioning scheme selected under the AIC criterion contained 8 data blocks (Additional file 3).

**Table 3:** Summary information of molecular data analyzed in this study

	Mitochondrial			Nuclear			
	16S	12S	COI	myh6	PTR	Rag 1	Rag2
Number of sequences	334	323	247	293	212	218	265
Length (bp)	574	429	522	621	537	1362	771
% present data	94	91	69	82	60	61	74
Number of variable sites	244	164	206	225	161	556	364
Singletons	46	29	4	21	22	100	84
Nucleotide frequency							
T	22.3	22.7	31.7	23.7	24.8	21.9	22.7
C	22.4	25.3	26	21.7	26.9	24.1	25.9
A	32.2	30.8	25	31.4	27.4	24.9	24.6
G	23.1	21.2	17.4	23.2	21	29.1	26.8
Overall mean genetic distance (p-value)	0.037 (±0.004)	0.051 (±0.006)	0.132 (±0.008)	0.011 (±0.002)	0.006 (±0.002)	0.031 (±0.007)	0.024 (±0.004)

DNA sequence information and composition of molecular markers used in this study. Overall mean genetic distance is an indication of the rate of evolution of each marker

**Fig. 3:**
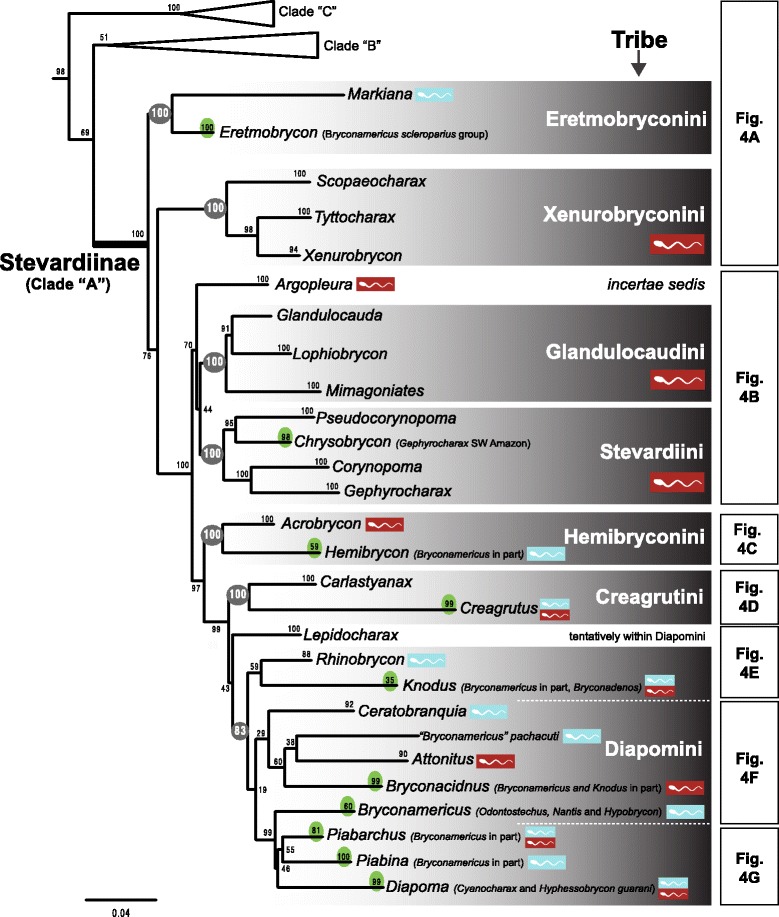
Abbreviated tree of Stevardiinae obtained in this study. Abbreviated maximum likelihood tree of Stevardiinae obtained with *RAxML* summarizing relationships among Stevardiinae genera and limits of tribes. Branches leading to monophyletic genera as proposed in this study, or to outgroup clades (clade “B” and “C”) are collapsed to a single terminal. Taxa currently assigned to other genera that are included in the proposed genera are indicated in parentheses. Red sperm symbols highlight clades with inseminating strategy present and blue sperm symbols highlight clades with external fertilization confirmed: large symbols for all taxa within Xenurobryconini, Glandulocaudini, and Stevardiini and smaller symbols for some species next to corresponding genera. Bootstrap values are shown for internal branches, with values inside gray circles highlighting nodes for proposed tribes and green circles showing support for monophyletic genera proposed in this study. Full topology is displayed in Figs. 4–10 and Additional file 4

**Fig. 4:**
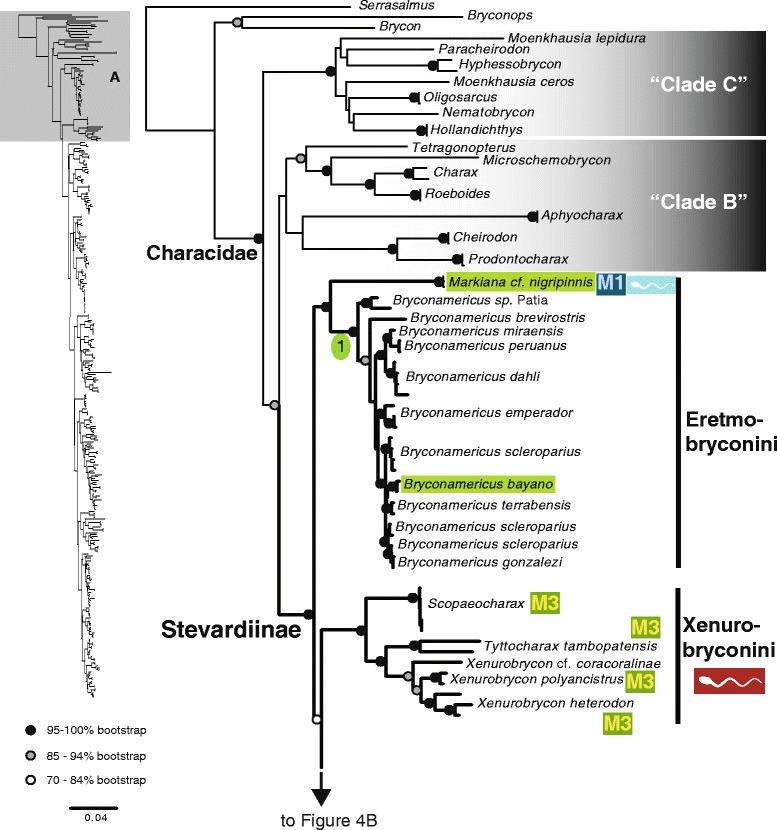
Section of the Stevardiinae phylogeny showing the relationships among clades “B” and “C” and Stevardiinae, and, within Stevardiinae, in the tribes Eretmobryconini and Xenurobryconini. ML tree obtained with *RAxML*. Single name labels several terminals when they lead to same nominal species. Type species of each genus are highlighted in green when sampled in this study. Red sperm symbols highlight taxa with insemination strategy, while blue sperm symbols highlight taxa known to have external fertilization. Sperm types are indicated by M1 – M3. Absence of any symbols next to taxon names indicates lack of knowledge about reproductive characters. Bootstrap values indicated with dots placed on internal branches according to inset caption. Section of the full topology shown on the left (shaded) is expanded on the right. Node 1 subtends *Eretmobrycon* (resurrected in this study; type species *E. bayano*) that includes all species of *Bryconamericus scleroparius* group (*Bryconamericus* species from Central America and northernmost South America)

**Fig. 5:**
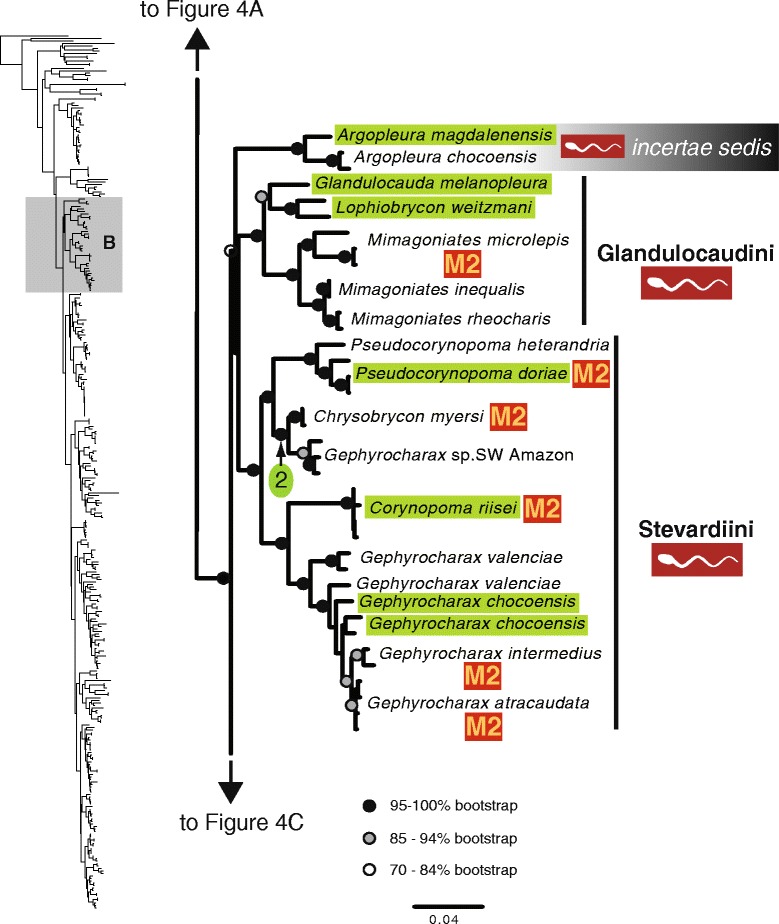
Section of the Stevardiinae phylogeny showing the relationships between *Argopleura* and the tribes Glandulocaudini and Stevardiini. ML tree obtained with *RAxML*. Single name labels several terminals when they lead to same nominal species. Type species of each genus are highlighted in green when sampled in this study. Red sperm symbols highlight taxa with insemination strategy, while blue sperm symbols highlight taxa known to have external fertilization. Sperm types are indicated by M1 – M3. Absence of any symbols next to taxon names indicates lack of knowledge about reproductive characters. Bootstrap values indicated with dots placed on internal branches according to inset caption. Section of the full topology shown on the left (shaded) is expanded on the right. Node 2 subtends *Chrysobrycon* from southwestern Amazon (type species *C. hesperus* was not available for this study)

**Fig. 6:**
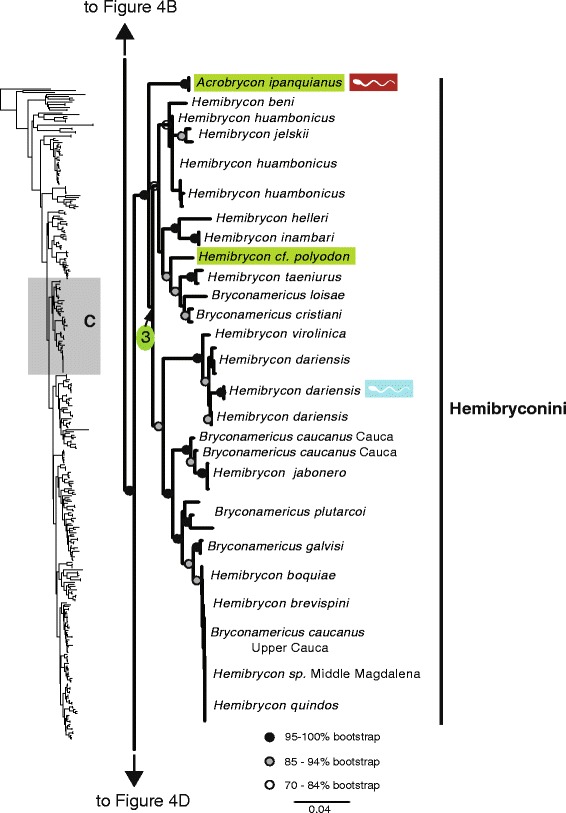
Section of the Stevardiinae phylogeny showing the relationships in Hemibryconini. ML tree obtained with *RAxML*. Single name labels several terminals when they lead to same nominal species. Type species of each genus are highlighted in green when sampled in this study. Red sperm symbols highlight taxa with insemination strategy, while blue sperm symbols highlight taxa known to have external fertilization. Sperm types are indicated by M1 – M3. Absence of any symbols next to taxon names indicates lack of knowledge about reproductive characters. Bootstrap values indicated with dots placed on internal branches according to inset caption. Section of the full topology shown on the left (shaded) is expanded on the right. Node 3 subtends *Hemibrycon* from western Amazon, Magdalena and Orinoco basins, and Central America (type species *H. polyodon*)

**Fig. 7:**
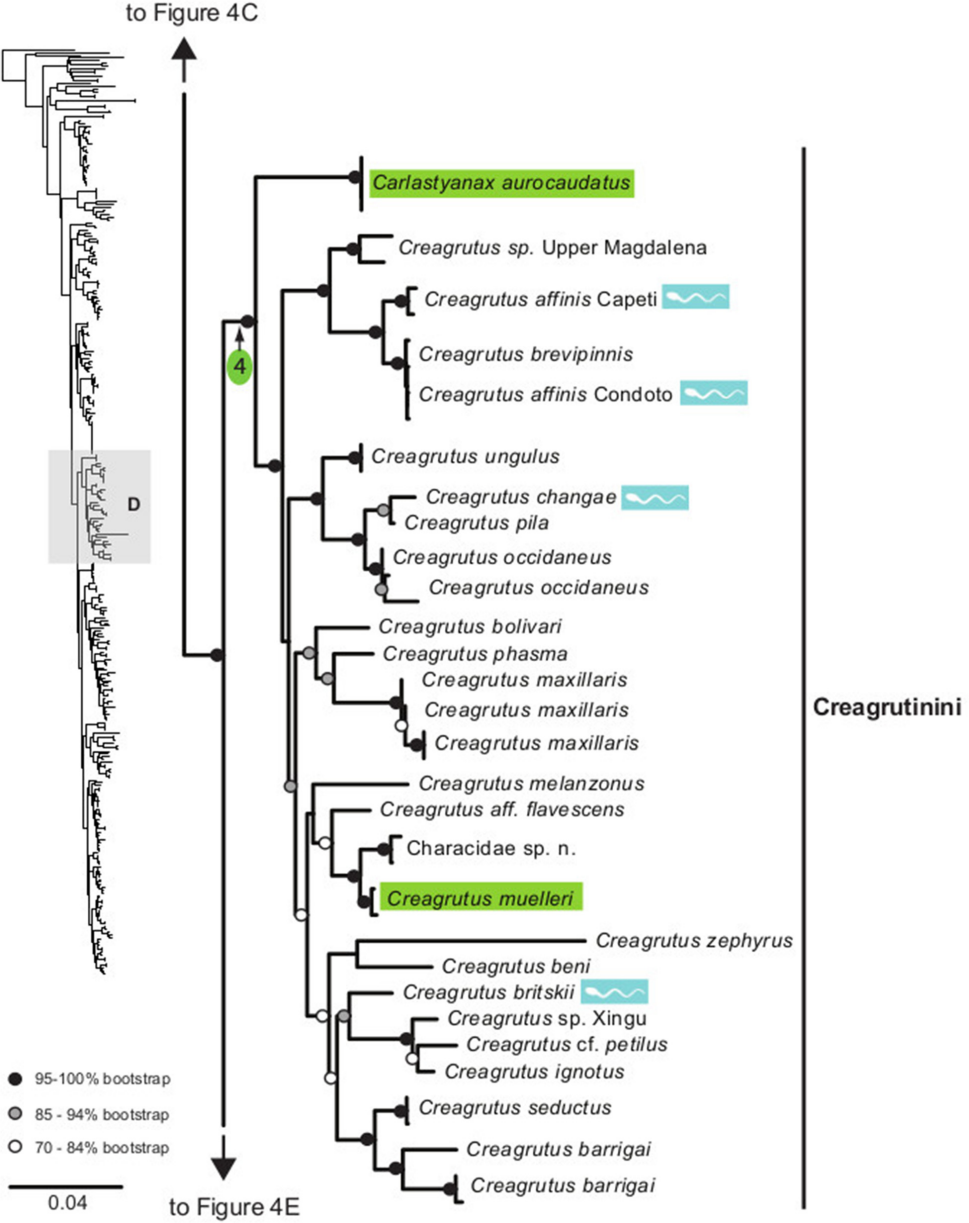
Section of the Stevardiinae phylogeny showing the relationships in Creagrutini. ML tree obtained with *RAxML*. Single name labels several terminals when they lead to same nominal species. Type species of each genus are highlighted in green when sampled in this study. Red sperm symbols highlight taxa with insemination strategy, while blue sperm symbols highlight taxa known to have external fertilization. Sperm types are indicated by M1 – M3. Absence of any symbols next to taxon names indicates lack of knowledge about reproductive characters. Bootstrap values indicated with dots placed on internal branches according to inset caption. Section of the full topology shown on the left (shaded) is expanded on the right. Node 4 subtends Creagrutini, with *Carlastyanax* (*C. aurocaudatus* is type species) and *Creagrutus* (*C. mulleri* is type species), the latter widely distributed from Paraguay basin to Central America

**Fig. 8:**
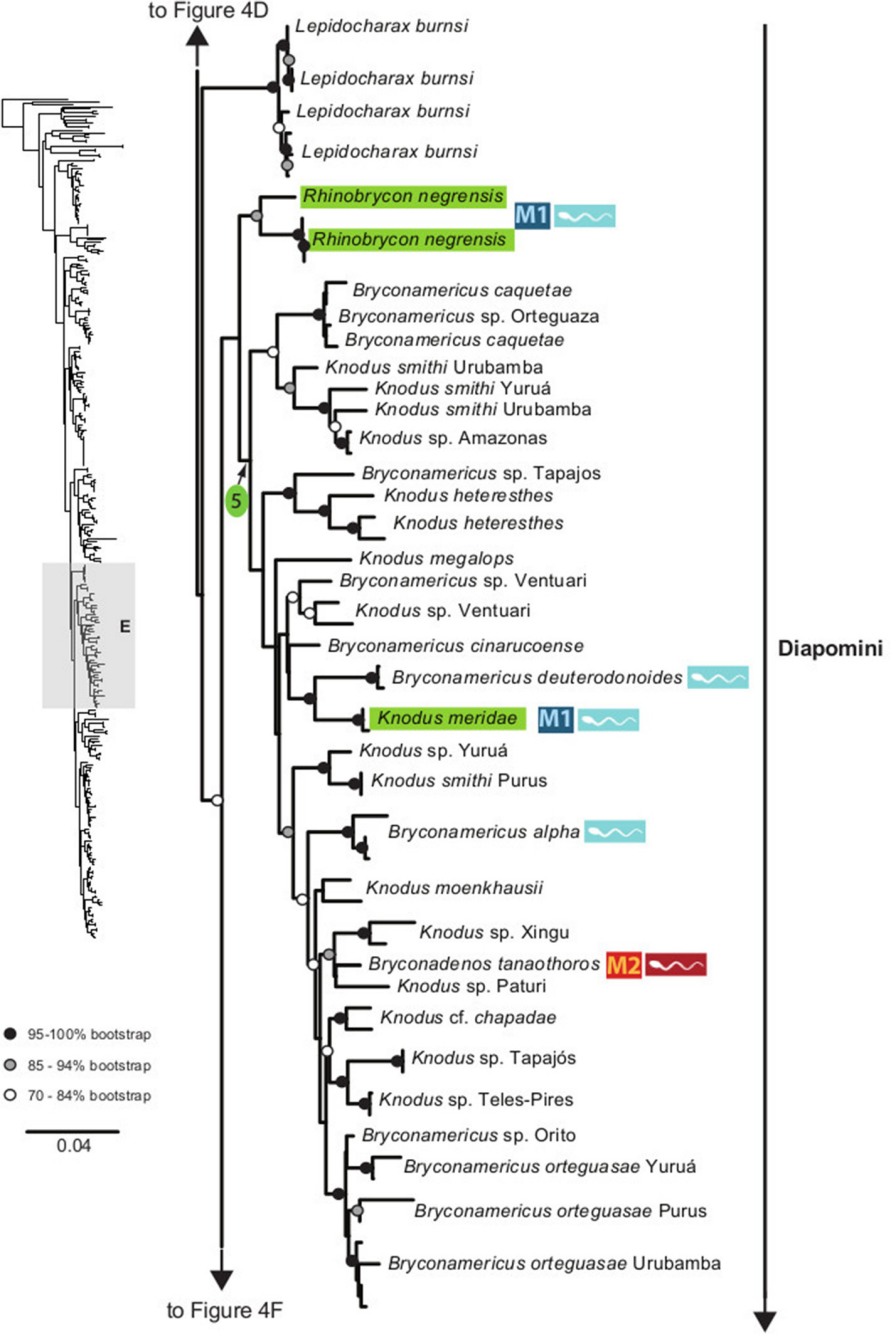
Section of the Stevardiinae phylogeny showing relationships in Diapomini (in part, continues in Figs. 9 and 10). ML tree obtained with *RAxML*. Single name labels several terminals when they lead to same nominal species. Type species of each genus are highlighted in green when sampled in this study. Red sperm symbols highlight taxa with insemination strategy, while blue sperm symbols highlight taxa known to have external fertilization. Sperm types are indicated by M1 – M3. Absence of any symbols next to taxon names indicates lack of knowledge about reproductive characters. Bootstrap values indicated with dots placed on internal branches according to inset caption. Section of the full topology shown on the left (shaded) is expanded on the right. Node 5 subtends *Knodus sensu stricto*, widely distributed in Amazon and Orinoco basins (*K. meridae* is type species)

**Fig. 9:**
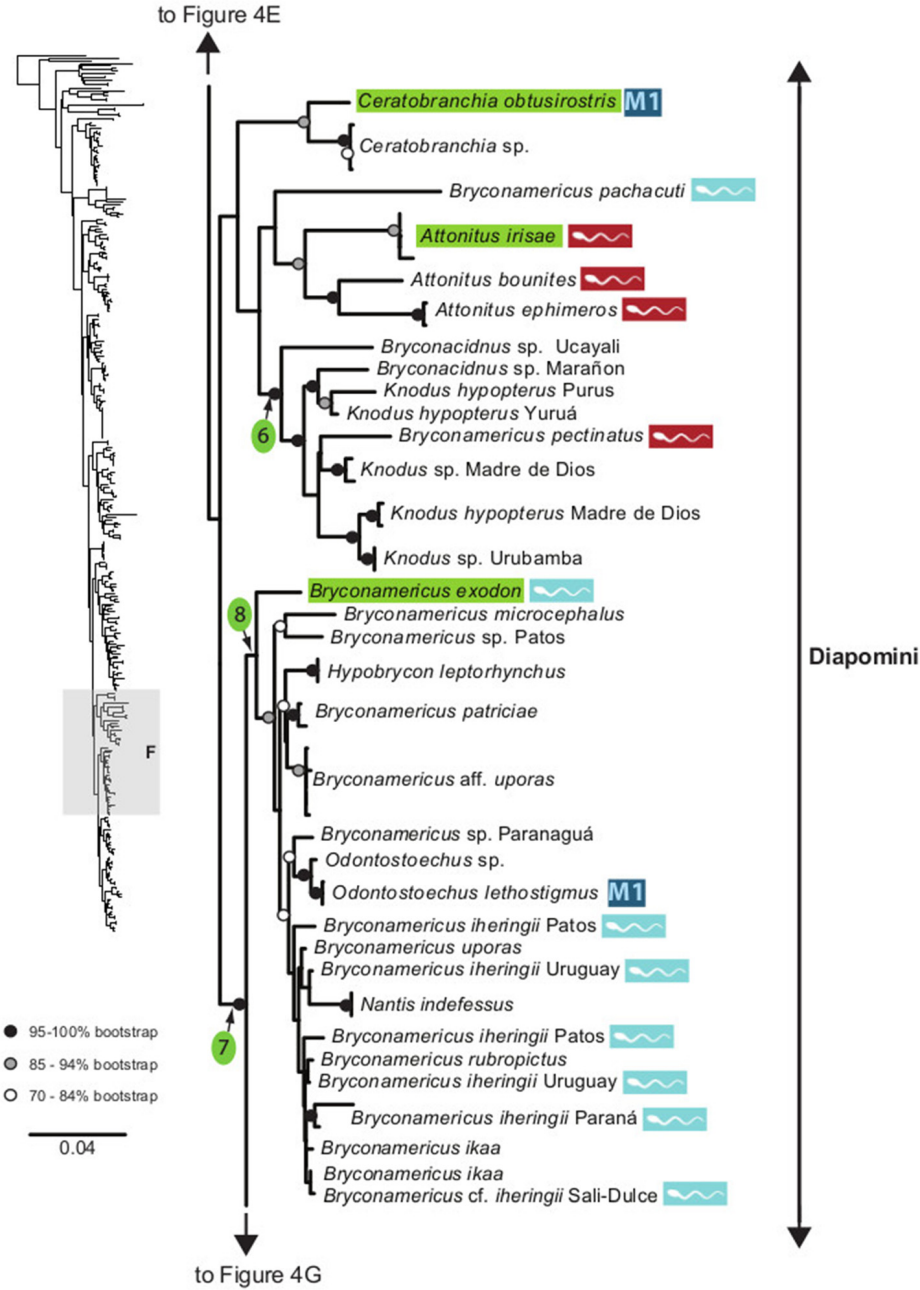
Section of the Stevardiinae phylogeny showing the relationships in Diapomini (in part, continues in Figs. 8 and 10). ML tree obtained with *RAxML*. Single name labels several terminals when they lead to same nominal species. Type species of each genus are highlighted in green when sampled in this study. Red sperm symbols highlight taxa with insemination strategy, while blue sperm symbols highlight taxa known to have external fertilization. Sperm types are indicated by M1 – M3. Absence of any symbols next to taxon names indicates lack of knowledge about reproductive characters. Bootstrap values indicated with dots placed on internal branches according to inset caption. Section of the full topology shown on the left (shaded) is expanded on the right. Node 6 subtends *Bryconacidnus* from western Amazon (type species *B. ellisi* non available for this study). Node 7 (continues in Fig. 10) subtends a clade from the southern range of the geographic distribution (Paraná, Paraguay, Uruguay, São Francisco and coastal basins in SE Brazil) that contains *Bryconamericus sensu stricto, Piabarchus, Piabina* and *Diapoma*. Node 8 subtends *Bryconamericus sensu stricto* restricted to Rio Paraná and Uruguay basins and coastal rivers in southeastern Brazil (*B. exodon* is type species)

**Fig. 10:**
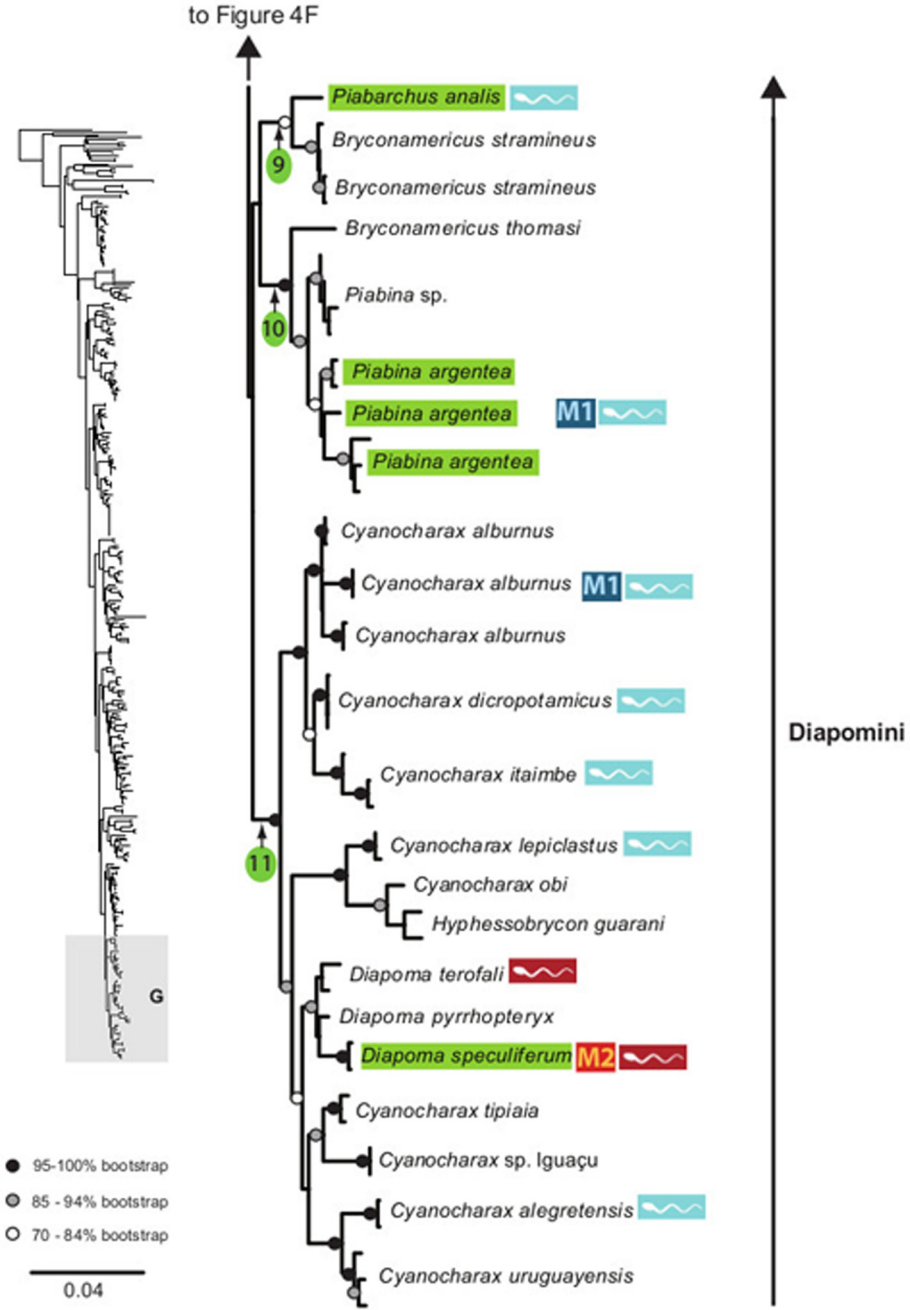
Section of the Stevardiinae phylogeny showing the relationships in Diapomini (in part, continues in Figs. 8 and 9). ML tree obtained with *RAxML*. Single name labels several terminals when they lead to same nominal species. Type species of each genus are highlighted in green when sampled in this study. Red sperm symbols highlight taxa with insemination strategy, while blue sperm symbols highlight taxa known to have external fertilization. Sperm types are indicated by M1 – M3. Absence of any symbols next to taxon names indicates lack of knowledge about reproductive characters. Bootstrap values indicated with dots placed on internal branches according to inset caption. Section of the full topology shown on the left (shaded) is expanded on the right. Node 9 subtends *Piabarchus* distributed in Rio São Francisco basin and upper Paraguay (*P. analis* is type species). Node 10 subtends *Piabina* from the Rio São Francisco, upper Paraná and Paraguay basins (*P. argentea* is type species). Node 11 subtends *Diapoma* from the Paraná, Uruguay and coastal systems in southern Brazil (type species *D. speculiferum*)

The maximum likelihood tree obtained with RAxML is shown in Fig. 3 in a summarized view, rendered by collapsing major clades to single terminals. The three large clades previously reported for Characidae (clades “A”, “B” and “C” [10, 12, 30]) are well-supported by the data, with “clade A” (representing the monophyletic subfamily Stevardiinae) resolved as the sister group of “clade B”. Seven clades with high bootstrap support were obtained within Stevardiinae, some in agreement with previous classifications but most clades are new. Fig. 3 presents a phylogenetic classification for the subfamily Stevardiinae and a proposed definition of monophyletic tribes and genera based on the taxonomic sampling analyzed in this study. The complete tree is available in detail in Figs. 4–10 and in Additional file 4. The molecular markers used in this study provided good phylogenetic resolution with high bootstrap support throughout the tree, with an average value of 78 % across branches and with more than half of the values equal to, or higher than, 90 %. *Landonia latidens* was excluded from the analysis since most genes for this taxon could not be amplified and sequenced, resulting in an unstable phylogenetic position for this species. Clades defining genera and other monophyletic groups within tribes received higher bootstrap support than branches leading to larger clades, especially clades containing the particularly species-rich genera such as *Bryconamericus* and *Knodus*. When comparing the stability among all trees accessed in this study, the newly circumscribed groups (tribes and genera) proposed herein are largely obtained by all methods with high support values (Table 4), except for the highly species-rich genera (*Bryconamericus*, *Hemibrycon* and *Knodus*) that received low support. The trees obtained with *Garli*, *TNT* and *STAR* are not shown, but some results from these analyses are reported in Table 4. All trees and data matrix obtained in this study are available at Dryad repository (doi:"http://dx.doi.org/10.5061/dryad.7nd42).

**Table 4:** Clade support obtained with different phylogenetic methods

Clades	Concatenation			Species tree
	RAxML	Garli	TNT	STAR
	(ML)	(ML)	(MP)	
Stevardiinae	100	100	99	yes^a^
Creagrutini	100	99	77	n/o
Diapomini	83	76	61	yes
Diapomini + *Lepidocharax*	43	97	19	n/o
Eretmobryconini	100	100	94	yes
Glandulocaudini	100	100	95	yes
Hemibryconini	100	99	82	yes
Stevardiini	100	100	98	yes
Xenurobryconini	100	100	99	yes
*Bryconacidnus* “clade”	99	94	81	yes
*Bryconamericus*, new circumscription	60	63	49	n/o
*Creagrutus*	99	98	78	yes
*Diapoma,* new circumscription	99	92	61	n/o
*Eretmobrycon*, new circumscription	100	100	86	yes
*Hemibrycon*, new circumscription	59	52	n/o	yes
*Knodus*, new circumscription	35	74	38	n/o
*Piabarchus*, new circumscription	81	80	74	yes
*Piabina*, new circumscription	100	100	99	yes

Support for clades representing tribes and genera proposed in this study (Fig. 3) obtained on the basis of maximum likelihood (*RAxML* and *Garli*), parsimony (*TNT*), and species-tree (*STAR*) analyses. Bootstrap values (%) are shown when the clade was obtained with maximum likelihood and parsimony, or indicated otherwise when it was not obtained (n/o). For the *STAR* analysis presence (yes) or absence (no) of the clade is indicated

^a^Some gene trees did not support the monophyly of Stevardiinae

The monophyly of genera *Bryconamericus*, *Cyanocharax*, *Hemibrycon*, and *Knodus* is strongly rejected by topology tests based on the molecular data (Table 5). This is also true for the tribes Diapomini and Xenurobryconini *sensu* Weitzman and Menezes [13] (Table 1). Based on the distribution of the species with known reproductive strategy and sperm morphology (Figs. 4–10), the hypothesis that inseminating stevardiines are monophyletic is rejected. Only sperm morphology M3 (restricted to *Scopaeocharax*, *Tyttocharax* and *Xenurobrycon*; Fig. 4) is inferred to have a single origin given that it is present within a monophyletic unit in our tree topology. Conversely, M2 appears in several clades (i.e., Glandulocaudini, Stevardiini, *Bryconadenos* and *Diapoma*) and may have multiple origins (Figs. 5, 8 and 10), and M1 is observed in species distributed in several distantly related clades: near the root of the tree (Eretmobryconini: *Markiana*) and also in *Ceratobranchia, Cyanocharax, Knodus meridae, Piabina* and *Rhinobrycon* (Figs. 4, 8–10).

**Table 5:** Topology tests for currently accepted taxonomic groups

Taxon (hypothesis)	Δ In L	AU	SH	KH
*Bryconamericus*	−2214	0.000001**	0**	0**
*Cyanocharax*	−34.4	0.021*	0.031**	0.031**
*Hemibrycon*	−536.5	0.00002**	0**	0**
*Knodus*	−570.8	0.000001**	0**	0**
Stevardiini	−34.1	0.1^*ns*^	0.1^*ns*^	0.1^*ns*^
Diapomini	−275.6	0.0000002**	0**	0**
Xenurobryconini	−158.9	0.00000003**	0**	0**
Inseminating species	−1004.6	0.000001**	0**	0**

Results of topology tests for the monophyly of currently accepted taxonomic groups (tribes and genera) and for groups based on reproductive strategy (insemination). Tribe composition tested prior hypotheses as shown in Table 1 [13, 19, 36], genera composition as listed by [2] and for insemination strategy as in Table 2. For each group, difference in maximum likelihood score (Δ lnL) between a tree with this clade enforced with respect to the maximum likelihood tree (Figs. 3–10) is followed by p-values for three testing procedures

*AU* approximately unbiased test; *SH* Shimodaira-Hasegawa test; *KH* Kishino-Hasegawa test

^*ns*^ *P* > 0.05; *P < 0.05; **P < 0.01

Based on the results presented here, we proposed a new classification of Stevardiinae, (see Additional file 5 – New Stevardiinae classification), subdividing the subfamily into seven tribes, redefining some generic circumscriptions, and leaving only 11 of the 48 genera as *incertae sedis*, as discussed below.

## Discussion

In agreement with previous molecular [7, 10, 12] and morphological studies [4, 8, 21], the monophyly of Stevardiinae or “clade A” [6] was resolved with confidence in our results (Figs. 3–10). The most comprehensive morphological analysis was based on 91 species in 20 genera [21] and a large-scale molecular phylogenetic analysis of the Characidae included 23 stevardiine species in 21 genera and along with a broad taxonomic representation of other subfamilies [12]. None of these previous studies, however, resolved internal relationships within Stevardiinae due to limited taxonomic sampling. Here we present analyses of a large and comprehensive sampling of Stevardiinae, with 32 genera and around 153 species/morphotypes, which identification was based on morphology and geographic location (see Additional file 2), and propose a new classification (Fig. 3, Additional file 5) based on monophyletic units (tribes and genera) supported by our results. The following sections address morphological, reproductive and geographic distributional data in relation to the major clades supported by this study, as well as limitations imposed by missing data and ambiguous phylogenetic resolution in subsections of the phylogeny.

### Monophyly of the Stevardiinae

Our results are consistent with the definition of “clade A” based on the presence of four teeth in the inner row of the premaxilla [6] (reversed to five teeth in *Nantis*), but not with the placement of *Bryconamericus scleroparius* clade and *Markiana* (positioned in *Astyanax* clade) outside the Stevardiinae [4, 8, 21] (Fig. 1b). This alternative hypothesis requires the separate origin of this trait in *Markiana* and in a clade containing the subfamilies Aphyocharacinae, Aphyoditeinae, Cheirodontinae, Gymnocharacinae, and Stevardiinae plus the *Bryconamericus scleroparius* clade, with a reversal to five or more teeth (in a single series) in a less inclusive clade containing Aphyocharacinae, Aphyoditeinae, and Cheirodontinae. The inclusion of the *Bryconamericus scleroparius* clade and *Markiana* in the Stevardiinae herein contradicts this hypothesis and resolves the presence of four teeth in the inner series of the premaxilla as a diagnostic character for the Stevardiinae (reversed to five teeth in *Nantis*).

A dorsal fin with ii+8 fin rays [6] is another diagnostic character proposed for “clade A”; although some authors split this trait into two characters [4, 8, 21]: eight or fewer branched dorsal-fin rays and nine or fewer dorsal-fin pterygiophores. According to our results (Figs. 3 and 4), the ii+8 dorsal-fin rays would not be a diagnostic character for Stevardiinae, but for a less inclusive clade including all Stevardiinae except *Markiana* plus *Bryconamericus scleroparius* group (the new tribe Eretmobryconini - see below). The inferred order of appearance of these diagnostic characters along the tree is informative as to the placement of two fossil species of *Paleotetra* from the Eocene-Oligocene (*P. entrecorregos* and *P. aiuruoca*) to support the hypothesis that these fossils constitute a stem lineage of Stevardiinae [3] or potentially within the tribe Eretmobryconini, since these fossils have four teeth in the inner series of the premaxilla but ii+9 dorsal-fin rays. Secondary increases in the number of dorsal-fin rays are recorded in *Pseudocorynopoma doriae* (ii+9), *Mimagoniates rheocharis* (ii+8-12), *Chrysobrycon myersi* (ii+9-10), differing from the other species of these genera with ii+8 dorsal-fin rays [6].

Seven strongly supported monophyletic groups observed in the phylogeny (Fig. 3), herein designated tribe-level taxa are discussed in the following sections. *Argopleura* is the only genus that could not be included in any tribe with confidence but it is closely related to Glandulocaudini and Stevardiini (as newly defined herein).

### Eretmobryconini, new tribe

The East Andean genus *Markiana* and the West Andean and Central American species of *Bryconamericus* (*B. bayano, B. brevirostris, B. dahli, B. emperador, B. gonzalezi*, *B. miraensis, B. peruanus, B. scleroparius,* and *B. terrabensis*) form a strongly supported clade (100 % boostrap) that is the sister group of the remaining Stevardiinae. They share with Stevardiinae the apomorphic presence of four teeth in the inner series of the premaxilla, but have ii+9 dorsal fin rays, which is considered a plesiomorphic state in Characidae.

*Markiana* is a monophyletic genus with two geographically disjunct species distributed in the Rio Orinoco and the Paraná, Paraguay and Mamoré river basins. Its relationships with other taxa were not clearly resolved and hence the genus was treated as *incertae sedis* in Characidae [2]. *Markiana* was first proposed as belonging to Stevardiinae [31] based on spermiogenesis, sperm morphology, the possession of four teeth in the inner series of the premaxilla, and short triangular ectopterygoid. Consistent with our results, this genus has been found related with the Central American characid species *Bryconamericus emperador* [12], currently assigned to the *Bryconamericus scleroparius* group [8, 21] and this clade formed the sister group to the remaining Stevardiinae. Although Mirande and collaborators [4, 8, 21] favored a grouping of *Markiana* with *Astyanax,* separately from the *Bryconamericus scleroparius* group and from the Stevardiinae, they found this relationship variable and with low stability in their analyses, and noted that *Markiana* and the *Bryconamericus scleroparius* group “share the absence of an ossified rhinosphenoid, an overlap of the horizontal arm of the preopercle by the third infraorbital, the possession of only four teeth on the inner premaxillary row, and the presence of two uroneurals” [21]. Under self-weighted parsimony optimization [8] and in other analyses [21], these characters support the monophyly of a group formed by these two taxa. Taken together, all the evidence strongly supports the recognition of *Markiana* plus the *Bryconamericus scleroparius* group as a monophyletic unit, and contradicts alternative hypotheses grouping *Markiana* and *Astyanax*.

The species of the *Bryconamericus scleroparius* group form a clade both in our hypothesis based on molecular data (node 1, Fig. 4: *B. bayano, B. brevirostris, B. dahli, B. emperador, B. gonzalezi*, *B. miraensis, B. peruanus, B. scleroparius, B. terrabensis,* and *B. sp.* from Río Patia) and on analysis based on morphological data [4, 8] (*B. brevirostris*, *B. emperador*, *B. peruanus*, *B. guaytarae*, *B. scleroparius*, and *B. simus*). These species are not closely related to *Bryconamericus exodon*, the type species of the genus, which is placed in a distant position in the phylogeny (node 8, Fig. 9), and must be recognized as a group separate from *Bryconamericus*. In addition, the monophyly of *Bryconamericus* is strongly rejected by our molecular data (Table 5). Among the species of *Bryconamericus scleroparius* group, *B. bayano* is the type species of the genus *Eretmobrycon* Fink, 1976, which was synonymized with *Bryconamericus* [32]. Based on our results and in previous results from morphology and sperm data [8, 21, 31], we propose the revalidation of the genus *Eretmobrycon* and the inclusion of all *Bryconamericus* species present in node 1 (*B. scleroparius* group; Fig. 4) within this genus (*E. bayano, E. brevirostris, E. dahli, E. emperador, E. gonzalezi*, *E. miraensis, E. peruanus, E. scleroparius,* and *E. terrabensis*). Also, *Bryconamericus guaytarae* and *B. simus*, not examined here, were tentatively resolved within the *B. scleoparius* group [4] and, because of it, are also tentatively included in *Eretmobrycon.* Even though the number of ii+9 dorsal-fin rays is plesiomorphic, it can be used as a further character to distinguish the species of *Eretmobrycon* from *Bryconamericus*.

### Xenurobryconini Myers and Böhlke, 1956, new usage 

The tribe Xenurobryconini, as currently defined, contains seven genera: *Argopleura*, *Chrysobrycon*, *Iotabrycon*, *Ptychocharax*, *Scopaeochrax*, *Tyttocharax* and *Xenurobrycon* (Table 1). This group was first proposed for *Xenurobrycon* and *Tyttocharax* [33] based on morphological similarities of their caudal fins. *Tyttocharax* was later split in two genera: *Tyttocharax* and *Scopaeocharax* [18], and in this same study *Argopleura* and *Iotabrycon* were added as members of the Xenurobryconini. *Ptychocharax* and *Chrysobrycon* were described later [13, 34] and also added to the tribe.

The monophyly of this tribe is strongly rejected by our molecular data (Table 5). Instead, our results (Figs. 3 and 4 and Table 4) resolved a highly supported clade (*Scopaeocharax* (*Tyttocharax + Xenurobrycon*)) congruent with “Subgroup B xenurobryconins” *sensu* Weitzman and Fink [18], diagnosed by 20 morphological synapomorphies. Monophyly of this group is further supported by the apomorphic sperm morphotype M3 shared by these three genera [11] (Table 2). *Iotabrycon* and *Ptychocharax* (not examined here) were found as successive sister groups to (*Xenurobrycon* (*Scopaeocharax* + *Tyttocharax*)) [18], but further investigation is necessary to test their membership within Xenurobryconini since molecular or sperm ultrastructure information are currently unavailable for these two genera. We provisionally list them within Xenurobryconini (Additional file 5).

The other two genera, *Argopleura* and *Chrysobrycon*, are more closely related to the tribes Glandulocaudini and Stevardiini (Fig. 5). In most of our phylogenetic results, *Argopleura* is resolved as the sister group of Glandulocaudini, however in the ML tree reconstructed in *RAxML* it is placed as sister group of Glandulocaudini and Stevardiini (70 % bootstrap). Since affinities of *Argopleura* with Glandulocaudini and Stevardiini are not clearly resolved and *Argopleura* could be potentially included in Gladulocaudini (pending further investigation), this genus is temporarily placed *as incertae sedis* in Stevardiinae. The new circumscription of the tribe Xenurobryconini is restricted to the genera *Scopaeocharax, Tyttocharax*, and *Xenurobrycon,* and possibly *Iotabrycon* and *Ptychocharax.*

### Glandulocaudini Eigenmann, 1914 *sensu* Menezes and Weitzman, 2009

The monophyly of the tribe Glandulocaudini including the genera *Mimagoniates* as sister group of *Lophiobrycon* and *Glandulocauda* is supported by our results (Fig. 5). The monophyly of Glandulocaudini was hypothesized [35] based on the presence of modified caudal peduncle squamation extending onto the caudal fin from the ventral region of the dorsal caudal-fin lobe and by the presence of modified club cells on the caudal organ, which probably secretes a pheromone during courtship. All the species in this group are inseminating. Relationships within Glandulocaudini, grouping *Lophiobrycon* as sister group to *Glandulocauda* receive high bootstrap support (Figs. 3 and 5), rejecting previous hypothesis that placed *Lophiobrycon* as the sister group of the other two genera in this clade [35, 36].

### Stevardiini Gill, 1858, new usage

Monophyly of a clade composed by the tribes Hysteronotini, Phenacobryconini (not analyzed herein), Stevardiini and Xenurobryconini has been proposed [13, 19] based mostly on the morphology and histology of the caudal organ. The molecular data resolved a monophyletic group including *Chrysobrycon* (considered part of Xenurobryconini [18]) with other taxa previously assigned to Stevardiini and Hysteronotini (Fig. 5). The Stevardiini (= Corynopomini *sensu* Weitzman and Menezes [13]; Table 1) is resolved as a strongly supported monophyletic group that includes *Corynopoma riisei* and the four species of *Gephyrocharax* (*Pterobrycon* was not examined; Table 5). This clade is the sister group of a clade that includes *Pseudocorynopoma* (one of the two genera of the Hysteronotini), *Chrysobrycon myersi,* and a characid from the southwestern Amazon (Madre de Dios, Ucayali and Yuruá basins; see [37]: figure of *Gephyrocharax* sp.; MUSM 33860, 38.3 mm SL) tentatively assigned to *Gephyrocharax*. Strong support of node 2 (Fig. 5) suggests that this form could be assigned to *Chrysobrycon*, with whom it also shares a distribution in western Amazonia, while other species assigned to *Gephyrocharax* are distributed in the Orinoco, Atrato, and Central American rivers, but further study may be necessary to fully resolve this issue.

In conclusion, we recognize an extended tribe Stevardiini that includes *Chrysobrycon* and *Pseudocorynopoma*, and possibly *Hysteronotus*, in addition to the three genera currently recognized in this tribe (*Corynopoma, Gephyrocharax,* and *Pterobrycon*). Further analysis of *Pterobrycon* and *Hysteronorus* is necessary to resolve of the final composition of this tribe.

### Hemibryconini Géry, 1966, new usage

The Hemibryconini was first introduced [38] to refer to a large group of characids consisting of *Boehlkea*, *Bryconacidnus*, *Bryconamericus*, *Ceratobranchia*, *Coptobrycon, Hemibrycon*, *Knodus*, *Microgenys*, *Nematobrycon*, *Piabarchus*, *Rhinobrycon*, and *Rhinopetitia*. We adopt the name but modify the circumscription to recognize a well-supported clade that includes solely *Acrobrycon*, *Boehlkea* and *Hemibrycon*. These genera are characterized by the presence of teeth along more than one-half the length of the dentigerous margin of the maxilla [39, 40], which may constitute a diagnostic morphological character for the tribe. Although we did not examine *Boehlkea* in this study, it is provisionally included in Hemibryconini, based on this morphological evidence.

Previous studies that addressed relationships within Stevardiinae using sexually dimorphic characters inferred a sister-group relationship between *Acrobrycon* and *Diapoma* plus *Planaltina* [13]. Other studies based on osteological and external morphological characters failed to support this relationship, suggesting instead that *Acrobrycon* is most closely related to *Mimagoniates*, *Pseudocorynopoma*, and *Diapoma* [4, 8]. Similarly, other morphological studies have suggested a close relationship between either *Boehlkea* and *Hemibrycon* [39] or *Bryconamericus* and *Hemibrycon* [32]. The sister-group relationship between *Hemibrycon* and *Bryconamericus* lacks morphological or molecular support, which is not surprising since the monophyly of *Bryconamericus* has been strongly rejected by several independent studies [4, 10, 12]. In contrast to previous studies, we find strong support for the sister group relationship between *Acrobrycon* and *Hemibrycon* (Fig. 6).

Under the phylogenetic hypothesis presented in Fig. 6 (node 3), *Hemibrycon* is paraphyletic, including five species originally described in *Bryconamericus* (*B. cristiani*, *B. caucanus*, *B. galvisi* and *B. plutarcoi*). These species are not closely related to *Bryconamericus exodon*, the type species of the genus, which is placed in a distant position in the phylogeny (node 8, Fig. 9), and, therefore, should be recognized as a group separate from *Bryconamericus*. We reassign these five species to *Hemibrycon* (Additional file 5). *Hemibrycon* is found in both sides of the Andes, with at least two separations, showing a peripheral distribution pattern within the Amazon basin [39].

Our results also highlight issues with delimitation of species in *Hemibrycon.* For instance, the clade from the upper Río Cauca basin includes three nominal species with negligible genetic differentiation: *H. boquiae*, *H. brevispini*, and *H. quindos* (Fig. 6). Based on these results and taking into account the lack of strong morphological evidence diagnosing these nominal species [41, 42], it is likely that *H. brevispini*, and *H. quindos* are junior synonyms of *H. boquiae.* The specimen from the upper Cauca labeled *Bryconamericus caucanus* is likely misidentified and should be included in *H. boquiae*. Other specimens assigned to *Bryconamericus caucanus* form a differentiated and well-supported clade with *H. jabonero.* Further study of this group is warranted.

### Creagrutini Miles, 1943, new usage

The affinities between *Carlastyanax* and *Creagrutus* were recently explored [21], and seven morphological synapomorphies were identified uniting these genera, with one of these being unique among characids (the presence of a ligament between the ascending process of the maxilla and the dorsal margin of the alveolar premaxillary ramus). This clade is well supported in our analysis (node 4, Fig. 7) that also confirms the re-validation of *Carlastyanax* at the rank of genus and as sister group to *Creagrutus* [21]. However, according to our results *Piabina* is not the sister group of *Creagrutus* or *Creagrutus* plus *Carlastyanax*, rejecting previous proposals [16, 21]. As in *Hemibrycon*, *Creagrutus* has a distribution to the two sides of the Andes. Nested among the Amazonian species of *Creagrutus,* the molecular data include an enigmatic taxon collected from the Río Marañón (Perú), identified at this time as Characidae sp. n. that differs from *Creagrutus* in having flattened multicuspids teeth on the premaxilla, as opposed to the massive teeth typical of *Creagrutus*. This taxon is closely related to *C. muelleri* (Fig. 7), another species from the western Amazon basins, strongly suggesting that it should be assigned to *Creagrutus*.

### Diapomini Eigenmann, 1909, new usage

A large clade including the remaining taxa in Stevardiinae is supported by the molecular data (bootstrap 83 %) and herein named Diapomini, differing radically from the current usage [13] (Table 1). Diapomini herein includes taxa assigned to *Attonitus*, *Bryconacidnus*, *Ceratobranchia*, *Cyanocharax, Diapoma, Hypobrycon*, *Knodus*, *Nantis*, *Odontostechus*, *Piabina*, *Piabarchus* and *Rhinobrycon,* in addition to a large number of species of *Bryconamericus* plus *Hyphessobrycon guarani*. Within this tribe there are three large clades of which two clades with an Amazon-Orinoco basin distribution are weakly supported and one clade with a southern South America distribution is highly supported (see below). In addition, *Lepidocharax* was found [20] as sister group of all former Stevardiinae [19], except *Landonia* and *Glandulocauda*. In our results, *Lepidocharax* is instead placed as sister group of Diapomini in all our phylogenetic results (except in *STAR* tree), although with low support (Table 4; Figs. 3 and 8). However, because of the consistency found among trees where *Lepidocharax* is sister group of the Diapomini, we tentatively assign *Lepidocharax* as member of this tribe, but we highlight the necessity of further investigation.

The species with Amazon-Orinoco distribution are split into two weakly supported clades (Figs. 8–9), both of which contain specimens assigned to *Knodus* interspersed with other taxa. The monophyly of this genus is strongly rejected by the molecular data (Table 5). The paraphyly of *Knodus* with some species of *Bryconamericus* was already pointed in a morphological study [43]. Consistent with this hypothesis, our results support the separation of this genus in two clades. In the first Amazonian clade, a number of *Bryconamericus* species from the Amazon and Orinoco basins (*B. caquetae, B. cinarucoenses, B. deuterodonoides, B. alpha and B. orteguasae*) are grouped in a clade with nearly all of the species of *Knodus* analyzed here (node 5, Fig. 8), including its type species *K. meridae,* and the inseminating *Bryconadenos tanaothoros* from the Rio Xingu. Although the node subtending this group (node 5) received low bootstrap support (35 %), it is supported in all our results (Table 4), suggesting that *Knodus sensu stricto* could be circumscribed to this clade (Fig. 3). *Bryconadenos* was hypothesized as closely related to *Attonitus* [19], but our results support the inclusion of *Bryconadenos* in the genus *Knodus* as defined above. Some species definitions within this clade, most notably *K. smithi* from the Río Purus should be revised, since it does not group with other taxa from the Urubamba and Yuruá rivers assigned to the same species. *Knodus sensu stricto* is the sister group of *Rhinobrycon negrensis* (Fig. 8).

In the second Amazonian clade, a well-supported group (node 6, Fig. 9) includes the inseminating *Bryconamericus pectinatus* along with *Knodus hypopterus*, *Knodus* sp. from Madre de Dios and Urubamba (Perú) and two unidentified species of *Bryconacidnus.* This clade (“*Bryconacidnus*” clade) does not include the type species of either *Knodus* (node 5) or *Bryconamericus* (node 8, Fig. 9; see below), and the strong support for this group suggests that all these species could be assigned to *Bryconacidnus*, pending further study that includes the type species of this genus (*B. ellisi*).

Relationships of *Bryconamericus pachacuti* from the upper Amazon in Peru remain somewhat uncertain but our results place it as the sister group to *Attonitus* (Fig. 9) with low bootstrap support (38 %), and separated from *B. exodon* by five nodes. Generic assignment of *B. pachacuti* cannot be resolved with confidence, but it is tentatively retained in “*Bryconamericus*” pending further study. *Ceratobranchia* and *Attonitus* are resolved as monophyletic with confidence and placed with low support as closely related to the “*Bryconacidnus”* clade (Fig. 9).

The remaining members of the Diapomini form a strongly supported clade (bs = 99 %: node 7, Fig. 9) that occupies the southern portion of the range of the Stervardiinae and includes the species of *Bryconamericus* analyzed in our study and not discussed so far, including the type species of the genus, *B. exodon*, and the species of the remaining genera listed for the Diapomini.

The type species of *Bryconamericus* is grouped with node 8 (Fig. 9). Although this node receives relatively low bootstrap support (60 %), it is present in all results obtained in this study (Table 4). The sister group to *Bryconamericus exodon* is a clade (bs = 90 %, Fig. 9) formed by several species of *Bryconamericus* (*B. iheringii*, *B. ikaa*, *B. lethostigma*, *B. microcephalus*, *B. patriciae*, *B. rubropictus*, and *B. uporas*), and other taxa assigned to three small genera *Hypobrycon*, *Nantis*, and *Odontostoechus*. While describing the genus *Hypobrycon*, it has been hypothesized that some of the species described in *Bryconamericus* (*e.g.*, *B. iheringii*) would be possibly more closely related to this new genus than to *B. exodon*, and that the definition of *Hypobrycon* could be expanded to include these species [44]. Our finding corroborates the grouping of *Hypobrycon* with *Bryconamericus iheringii* and some other congeners but the recognition of *Hypobrycon* or of the monotypic genera *Nantis* and *Odontostoechus* as valid genera would demand the recognition of several small genera in node 8. *Odontostoechus* has been described originally in Cheirodontinae based on the presence of a single series of teeth in the premaxilla, but this seems to be an autapomorphy of the type species, *O. lethostigmus.* Similarly, the diagnostic characters of *Nantis* seem to constitute autapomorphies of the type species. Given that *Bryconamericus* has priority over all other nominal genera in this clade, our results provide the basis for defining a monophyletic genus *Bryconamericus sensu stricto* that includes all the species subtended by node 8 (Fig. 9). Further study of this group is warranted given the morphological diversity of the studied species. Other species of *Bryconamericus* not subtended by node 8 and not discussed in this study, would be retained in “*Bryconamericus*” until further study may justify shifting then to other genera (Additional file 5).

*Piabarchus analis* plus *Bryconamericus stramineus* (node 9, Fig. 10) and *Bryconamericus thomasi* plus *Piabina* (node 10, Fig. 10) form two well-supported groups. These results suggest that the two species assigned to *Bryconamericus* should be reassigned to *Piabarchus* and *Piabina*, respectively (Fig. 10), since they are only distantly related to the type species *Bryconamericus exodon*. A specimen from the Amazon basin in Peru assigned to *Piabarchus analis* [ROM 55742, 300 m E of Panguana camp, isolated pool of a Llulapichis river tributary, Peru, Huanuco, Ucayali River drainage, collected by Erling on 23 July 1988] was misidentified and corresponds to *Gephyrocharax* (Lopéz-Fernandéz, Taphorn, and Vanegas-Rios, pers. comm.). Therefore the distribution of *Piabarchus* is restricted to the Paraguay-Paraná and São Francisco basins.

Among the remaining genera included in this tribe (node 11, Fig. 10), there is a very well supported clade embracing *Cyanocharax*, *Diapoma* and “*Hyphessobrycon*” *guarani.* This clade is also supported by the apomorphic number of i+6 pelvic-fin rays, a count also shared with the species of *Planaltina* not analyzed herein, and differing from the other genera in Stevardiinae that have i+7 rays [6, 45–47]. The genus *Diapoma* (with all inseminating species) has been hypothesized as closely related to other inseminating species of Stevardiinae that also bear a caudal organ [13, 20, 35]. All these studies, however, did not include a comprehensive sampling of other species without caudal organs to test this relationship. It is of note that the caudal organs of the Diapomini [13] differ from those of taxa in other tribes in being present and identical in both males and females, while it is sexually dimorphic (only males) in other inseminating tribes (except in *Acrobrycon* [13]), suggesting the non-homology of the caudal organ of the Diapomini relative to other tribes. Our results place *Diapoma* as a clade inserted in *Cyanocharax*, making this genus as defined [6] paraphyletic. Similarities between *Cyanocharax* and *Diapoma*, such as tooth arrangement, general body shape, color pattern and number of pelvic-fin rays have been previously discussed [45], in a comparison of the morphology of *Diapoma speculiferum* and *Diapoma terofali* with *Cyanocharax alburnus* (therein named *Astyanax hasemani*). Based on the strong molecular support for this clade (node 11, Fig. 10), species of *Cyanocharax* and *Hyphessobrycon guarani* should be reassigned to the genus *Diapoma* (Additional file 5) in order to define monophyletic genera and to be consistent with a phylogenetic classification. *Diapoma* as herein defined includes five highly supported internal clades (bootstrap 99 %, 95 %, 85 %, 93 % and 99 %). Further study of this group is warranted given the morphological diversity of the included species and the presence of four well-defined internal lineages.

### Monotypic tribes (not analyzed herein)

Two monotypic tribes in Stevardiinae were not analyzed here: Landonini Weitzman and Menezes, 1998 and Phenacobryconini Weitzman and Menezes, 1998. *Landonia latidens* was sampled in our study, but excluded from our analyses because of few genes amplified that produced instability in the phylogeny; *Phenobrycon henni* was not available. As already pointed in the literature [48], the large amount of monotypic genera being proposed in Characidae to accommodate autapomorphies is somehow arbitrary and, in many instances, lacking phylogenetic inference. In the case of Landonini and Phenacobryconini, monotypic tribes were proposed to accommodate the large amount of autapomorphies presented by these monotypic genera; however these propositions do not reflect phylogenetic relationships. Since the relationships for these two monotypic genera are unknown in Stevardiinae, we placed them as *incertae sedis* (Additional file 5) to emphasize the necessity of further investigation of their relationships.

### Insemination

External fertilization is the most common reproductive strategy across the Ostariophysi, including Characiformes. Insemination as a reproductive strategy is found only in some representatives of the Characidae, including several members of the Stevardiinae (Table 2). Historically, the study of insemination was prompted by the presence of modified glandular organs in the caudal or anal fins of some species and documented histologically by the presence of sperm in the ovaries of stevardiine species that bear this caudal organ. Insemination, however, has also been documented for a few species lacking modified caudal or anal fins (e.g., *Bryconamericus pectinatus*, *Creagrutus lepidus* and *C. melasma*), but information on insemination, or the lack of it, is absent for most of the remaining members of this subfamily, being available only for 92 of the 311 species of Stevardiinae (Table 2), and only for 46 species analyzed here (30 %). Apparently due to the fact that external fertilization is ancestral and widespread in Characidae, characid species whose reproductive strategy is unknown have been treated informally in the literature as externally fertilized, but absence of evidence should not be used as evidence for absence. A topology test constraining all known inseminating taxa into one clade, however, rejects a single origin for insemination (Table 5). All members of Xenurobryconini, Glandulocaudini, Stevardiini and scattered species in Hemibryconini, Creagrutini and Diapomini are inseminating. This phylogenetic distribution suggests multiple origins and/or multiple losses of the inseminating reproductive strategy in Stevardiinae. That is in agreement with the general phylogenies of the Characidae [4, 7, 10, 12, 21]. Further analyses should produce more conclusive results once the reproductive strategy of most species of stevardiines is known.

In term of sperm morphology, M3 spermatozoid from the xenurobryconins is the only sperm morphology recovered as monophyletic, while M1 and M2 are found scattered along the tree. Since M1 appears close to the root in *Markiana* and in several other terminal nodes, it could constitute a diagnostic character for the Stevardiinae. For M2, the sporadic position for the few taxa that present this sperm type along the tree may indicate that this trait has multiple origins in Stevardiinae. However, the proposition of these sperm morphotypes was based on grouping multiple characters (e.g., centriole and midpiece position and slightly to moderately elongated shape) into a single sperm morphotype [11]. A reductionist analysis that creates artificial sperm types could lump independent characteristics, with distinguished evolutionary histories, into a single sperm group (e.g. M1, M2), not necessarily recovering phylogenetic relationships among taxa that share them.

Unfortunately, it is important to highlight that the lack of information about sperm morphology and reproductive strategy in several taxa precludes the use of probabilistic approaches for ancestral state reconstruction to test the hypotheses presented here.

## Conclusions

The molecular phylogeny presented in this study provides a significant advance in our knowledge of the relationships among of characid fishes in the subfamily Stevardiinae. On the basis of well-supported monophyletic groups, we defined seven tribes (Figs. 3–10, Additional file 5) and propose new circumscriptions for historically problematic (polyphyletic) genera such as *Bryconamericus* and *Knodus*, splitting part of the species of *Bryconamericus* into different and not closely related genera (*e.g.*, *Eretmobrycon* and *Hemibrycon*). Some key taxa not included in our study or with poor resolution in our phylogeny remain with uncertain classification, and point to necessary future studies. Reproductive traits among stevardiine species that include inseminating strategy and variable sperm morphology are interpreted under the new phylogenetic hypotheses to show that some are evolutionary labile characters while others are phylogenetically informative requiring additional documentation and further analyses to understand their origins and phylogenetic distributions.

## Methods

### Taxonomic sampling

A total of 330 specimens were obtained from species assigned to the subfamily Stevardiinae. These represent 153 species or morphotypes (49 % of 311 valid species in Stevardiinae), and 32 (67 %) of the 48 recognized genera, plus one species (*Hyphessobrycon guarani*) not currently included in Stevardiinae [6, 49]. These samples originated in all major Neotropical river basins. In addition, 25 samples from 17 characiform species were used as outgroup. All specimens used for this molecular work have tissue samples preserved in ethanol associated with voucher specimens in museum fish collections and were identified to species (or genus) based on diagnostic morphological traits. In cases that fieldwork was required, specimens collection was authorized by the Ministério do Meio Ambiente - MMA, Instituto Chico Mendes de Conservação da Biodiversidade - ICMBio, licence # 12038/2, in accordance with protocols in their ethical and methodological aspects for the use of fish. Specimens were euthanized with Eugenol after capture, and all efforts were made to minimize suffering. In addition, for two species sequences were obtained from GenBank (*Pseudocorynopoma heterandria* and *Piabarchus* sp. [12]). A complete list of tissues with their associated voucher identification, collection locality (basin) and contributing institutions (abbreviations as in [50]) is presented in Additional file 2.

### Molecular methods

Genomic DNA was extracted from muscle and fin tissues preserved in 96 % ethanol. For each specimen, DNA was extracted from approximate 20 to 30 mg of the tissue using the DNeasy tissue extraction kit (Qiagen) or Promega Wizard® SV 96 Genomic DNA Purification System. We collected DNA sequences from seven molecular markers (four nuclear: *MYH6, PTR, RAG1* and *RAG2*; and three mitochondrial genes: *16S, 12S* and *COI*).

PCR reactions to amplify mtDNA fragments used the Promega GoTaq® qPCR Master Mix in 30 μl reactions with the following concentrations: 10 to 50 ng genomic DNA, 0.16 mM of each primer, 0.9 mM of each dNTP, 1x PCR Buffer, 4 mM MgCl_2_ and 0.026 U Taq DNA polymerase. For the amplification of the nuclear loci, a nested-PCR approach was required using the Takara® LA PCR Kit. PCR reactions consisted of 10 to 50 ng genomic DNA, 2.5 μl of dNTP mix (1 mM each), 3.0 μl 10x buffer, 0.5 μl of each primer (10 μM), 1.2 μl of bovine serum albumin (BSA), 0.2 μl of Takara Taq (5 U/μl), and dH_2_O to a final volume of 30 μl. A list of the primers used as well as optimized PCR conditions for the seven markers are presented in the Additional file 6. PCR products were submitted for purification and sequencing in both directions to the High Throughput Sequencing facility, University of Washington, Seattle – Washington, USA. The forward and reverse chromatograms were assembled and visualized using the program *Codon Code Aligner* v3.7.1. (Codon Code Corporation). IUPAC ambiguity codes were applied when heterozygotes or uncertainty of the nucleotide identity was detected. All sequences produced for this study have been deposited [GenBank: KF209375 - KF209697 (*12S*), KF209698 - KF210029 (*16S*), KF210030 - KF210276 (*COI*), KF210277 - KF210567 (*MYH6*), KF210568 - KF210779 (*PTR*), KF210780 - KF210995 (*RAG1*), KF210996 - KF211258 (*RAG2*)] – see Additional file 2 for details.

### Phylogenetic analysis

The *16S* and *12S* sequences were aligned in *SATé* v1.4 [51] using 50 iterations under default settings. Exon markers and *COI* were aligned individually using *Muscle* v3.6 [52] software under the default parameters. All exons and *COI* alignments were unproblematic because these are conserved markers that exhibit very little or no length variation among species, but were, nevertheless, visually inspected using *Mesquite* v2.7 [53] to verify that all sequences follow the correct reading frame and contain no stop codons. Because nested PCR is highly prone to cross contamination, a quality control step that involved gene tree estimation via NJ was included in our workflow to detect potential cases of sequencing errors due to contamination. Given the degree of redundancy designed in our taxonomy sampling, errors due to either sequencing or misidentification can be detected when sequences from putative conspecific specimens are not placed together in the tree. Sequences that were found misplaced in the resulting gene trees were re-checked and re-sequenced or removed from the alignments in cases of poor quality. Once individual gene alignments were finalized, these were used for gene tree estimation (for species tree analysis, see below), and concatenated in a single matrix of all genes for phylogenetic analysis.

Phylogenetic reconstructions were performed using the concatenated dataset as well as species-tree approach that reconcile discordant gene genealogies. Concatenated analyses were conducted under Maximum Likelihood (ML) and Maximum Parsimony (MP) criteria. For ML analyses, the concatenated data matrix was partitioned into data blocks to account for heterogeneity among sites and to select the most appropriate partitioning scheme and models. *PartitionFinder* v1.1.1 [54] was implemented with data blocks defined *a priori* according to commonly used structural and functional criteria, separating the sequence data into 16 different blocks as follows: the mitochondrial *12S* and *16S* genes (one block) and each codon position of each protein-coding gene (*COI, MYH6, PTR, RAG1, RAG2*) for the other 15 blocks (3 codon positions x 5 genes). *PartitionFinder* tests combinations of the 16 initial subsets into a smaller number of data blocks to select the optimal partitioning scheme based on AIC scores and determine the best-fit model for each partition. Sequences of *Serrasalmus,* the most external characiform in our dataset, were used to root the phylogenetic analyses [12].

The ML analyses were run in *RAxML* v7.2.8 [55] and *Garli* v2.0 [56]. The evolutionary model used for all data blocks in *RAxML* was GTRGAMMA, while the *Garli* settings were adjusted according to the best-fit models selected with *PartitionFinder. RAxML* searches were conducted in the *CIPRES* portal v3.1 [57] using ten parallel runs and starting with a randomly generated tree. Branch support was assessed using the rapid bootstrap algorithm with 1000 replicates. *Garli* searches used automatic termination (enthreshfortopoterm command), with eight parallel runs, eight search replicates and 1000 bootstrap replicates.

For comparison purposes, the concatenated dataset was analyzed under equally weighted parsimony in *TNT* v1.1 [58]. The *TNT* analysis used a driven-search strategy combining several tree-search algorithms (*e.g.*, ratchet, drift, sectorial searches and tree fusion). To maximize tree-space exploration, the final searches implemented the tree-bisection–reconnection (TBR) algorithm with 1000 independent replicates. Assessment of branch support was based on bootstrap search strategies using TBR and 1000 replicates.

Species tree analyses were conducted in the program *STAR*, as implemented in the *STRAW* web server [59–61]. Input gene trees for *STAR* were estimated in *RAxML*, using the same settings as explained above, and re-rooted with *Serrasalmus*. The genes *CO1*, *12S*, and *16S* were concatenated and analyzed as a single mitochondrial locus; the four nuclear gene alignments were run separately, to obtain a total five gene trees for the *STAR* analysis. Preliminary analyses on three genes (*MYH6*, *RAG1*, and *PTR*) resulted in the non-monophyly of Stevardiinae. The ingroup monophyly was thus enforced and new gene trees were estimated in *RAxML*. Species that had sequences from multiple individuals were annotated using the Species Allele Table Creator in *STRAW*. Additional species tree analysis was performed using *BEAST 1.8.0 [62, 63] with one billion MCMC iterations, however this analysis did not reach convergence and for this reason are not reported here.

Finally, topology tests were conducted to assess whether the monophyly of traditionally recognized tribes and genera of Stevardiinae (that were not obtained in our resulting trees) can be rejected by the new data. We used *RAxML* to obtain maximum likelihood phylogenies consistent with the alternative hypotheses. The unconstrained ML tree, commonly leading to non-monophyly of the group of interest, was compared to the ML topology consistent with enforcing the monophyly of each of the tribes and genera challenged by our results. For each of these analyses, the best tree of 10 independent searches was selected. To evaluate the differences in likelihood scores between constrained and unconstrained tree topologies, the site likelihood scores were extracted using *RAxML* and various topological tests were performed in *Consel*, including the AU (approximately unbiased), SH (Shimodaira and Hasegawa), and KH (Kishino and Hasegawa) tests [64].

## Availability of supporting data

The data sets supporting the results of this article are available in the Dryad Digital Repository: http://datadryad.org/review?doi=doi:10.5061/dryad.7nd42 [65].

## Additional files

Additional file 1:
**Nomenclatural remarks regarding Stevardiinae, Glandulocaudinae and Corynopomini.**
Additional file 2:
**List of material examined, catalog numbers, GenBank accession numbers, locality and genetic coverage for each taxon collected in this study.**
Additional file 3:
**Best-fit models and partitioning scheme obtanied with PartitionFinder.**
Additional file 4:
**Unabridged ML tree obtained with RAxML.**
Additional file 5:
**New Stevardiinae classification based on phylogenetic relationships.**
Additional file 6:
**List of primers used and optimized PCR conditions for all markers.**
