# Erratum to: Molecular phylogeny of the subfamily Stevardiinae Gill, 1858 (Characiformes: Characidae): classification and the evolution of reproductive traits

**DOI:** 10.1186/s12862-015-0489-8

**Published:** 2015-12-03

**Authors:** Andréa T. Thomaz, Dahiana Arcila, Guillermo Ortí, Luiz R. Malabarba

**Affiliations:** Department of Ecology and Evolutionary Biology (EEB), University of Michigan, 1109 Geddes Ave., Ann Arbor, 48109 MI USA; Departamento de Zoologia, Universidade Federal do Rio Grande do Sul (UFRGS), Av. Bento Gonçalves 9500, Porto Alegre, 90501-970 RS Brazil; Department of Biological Sciences, The George Washington University, 2023 G St. NW, Washington, 20052 DC USA; Department of Vertebrate Zoology, National Museum of Natural History Smithsonian Institution, MRC 159, Washington, 20013 DC USA

## Erratum

The original version of this article unfortunately contained some mistakes. The presentation of Figs. 3–10 were incorrect in the HTML and PDF versions of this article. The corrected figures are given below. In addition, Additional file 4 and 5 were presented incorrectly. The updated versions of these files are also supplied below.

**Fig. 3:**
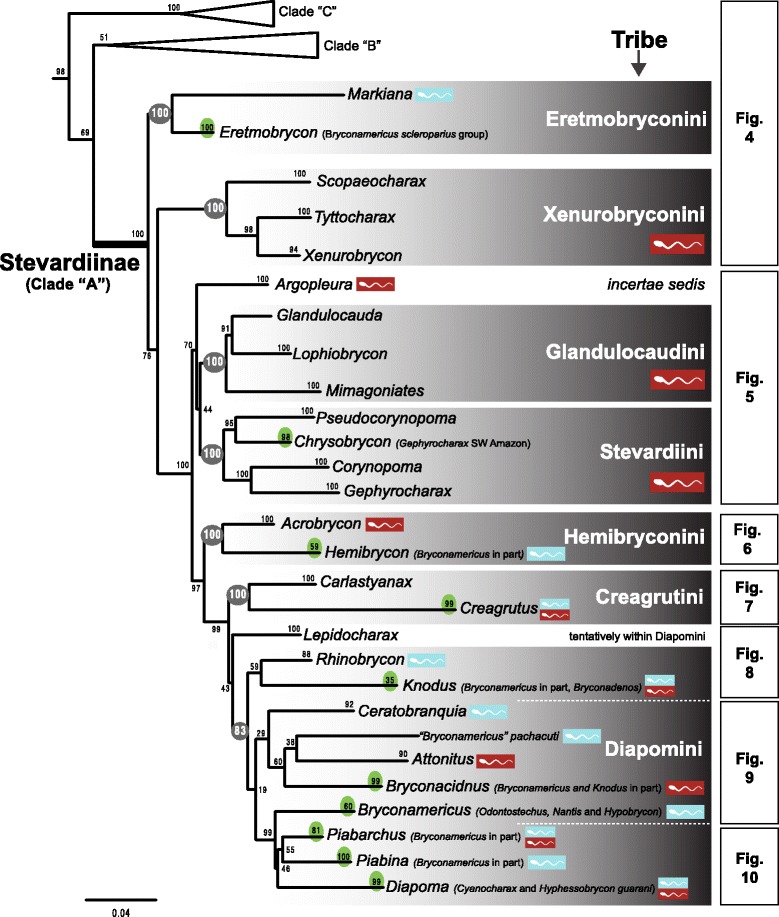
ᅟ

**Fig. 4:**
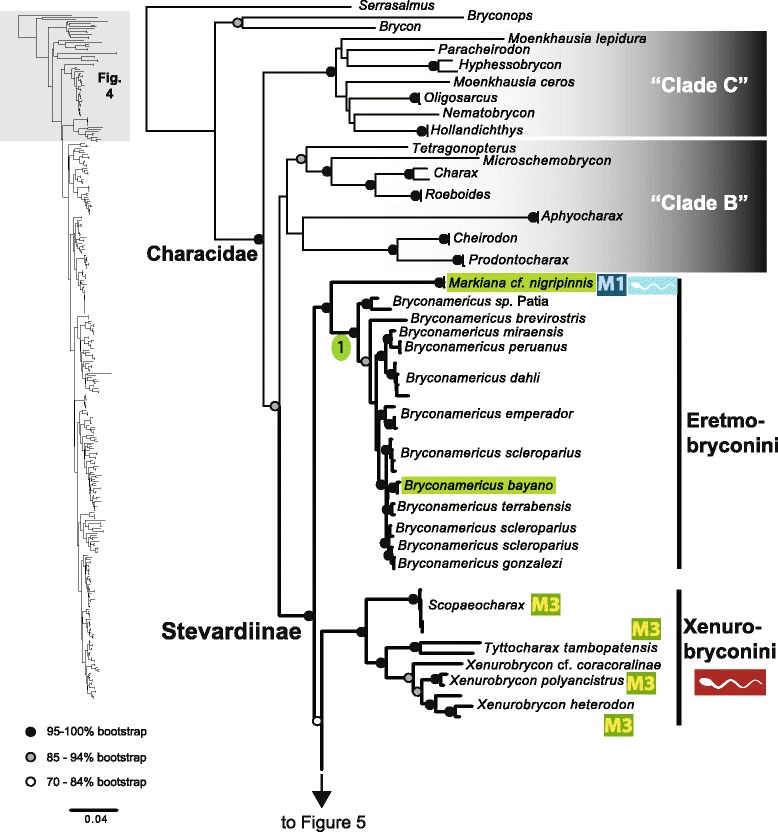
ᅟ

**Fig. 5:**
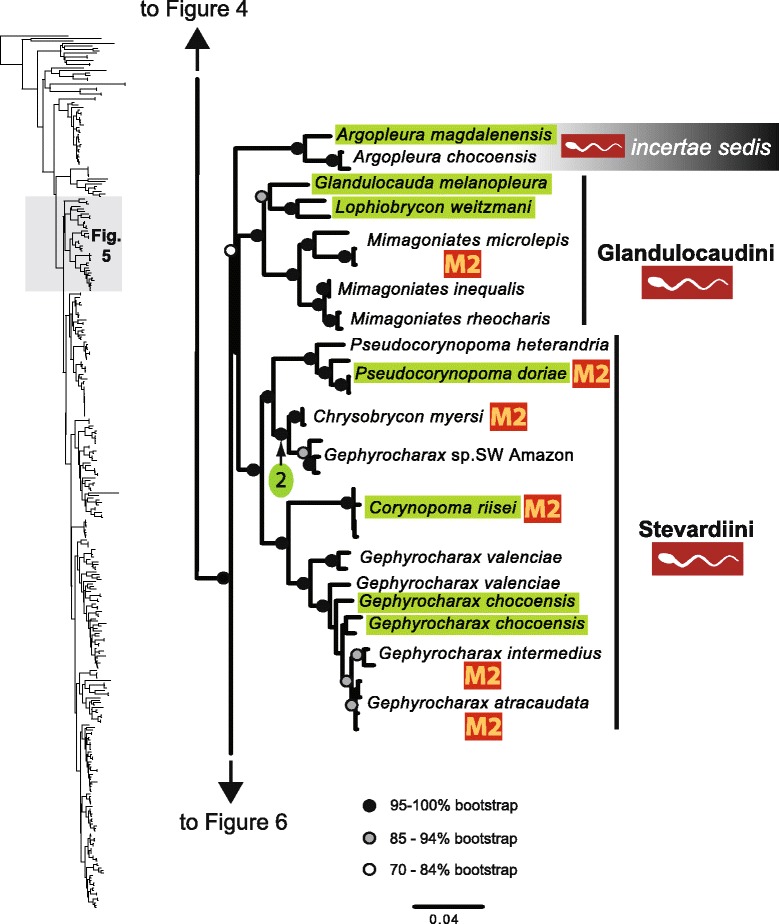
ᅟ

**Fig. 6:**
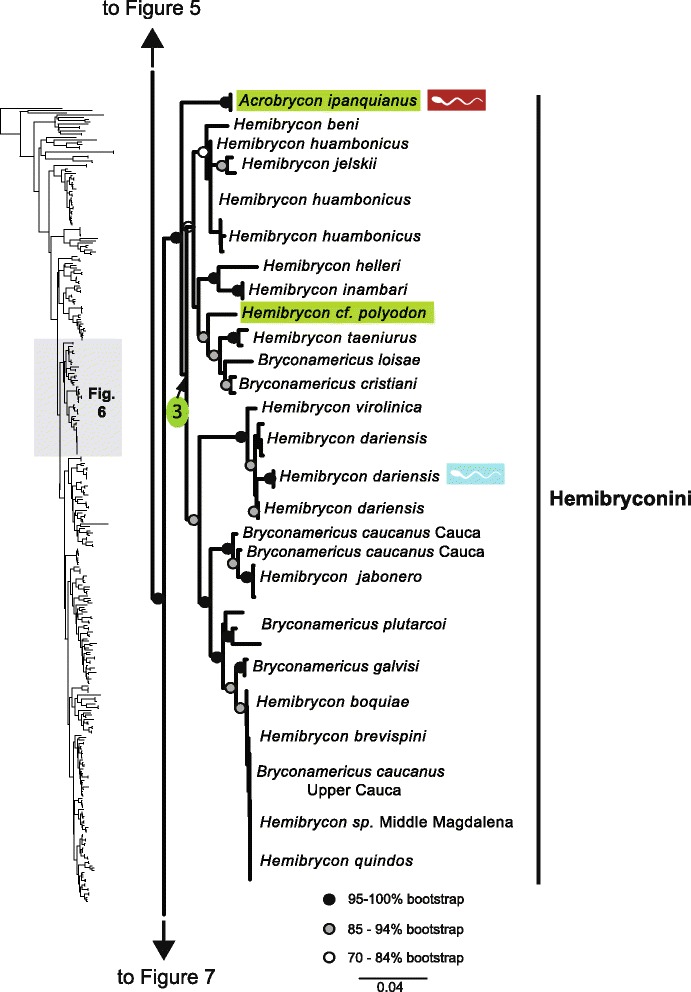
ᅟ

**Fig. 7:**
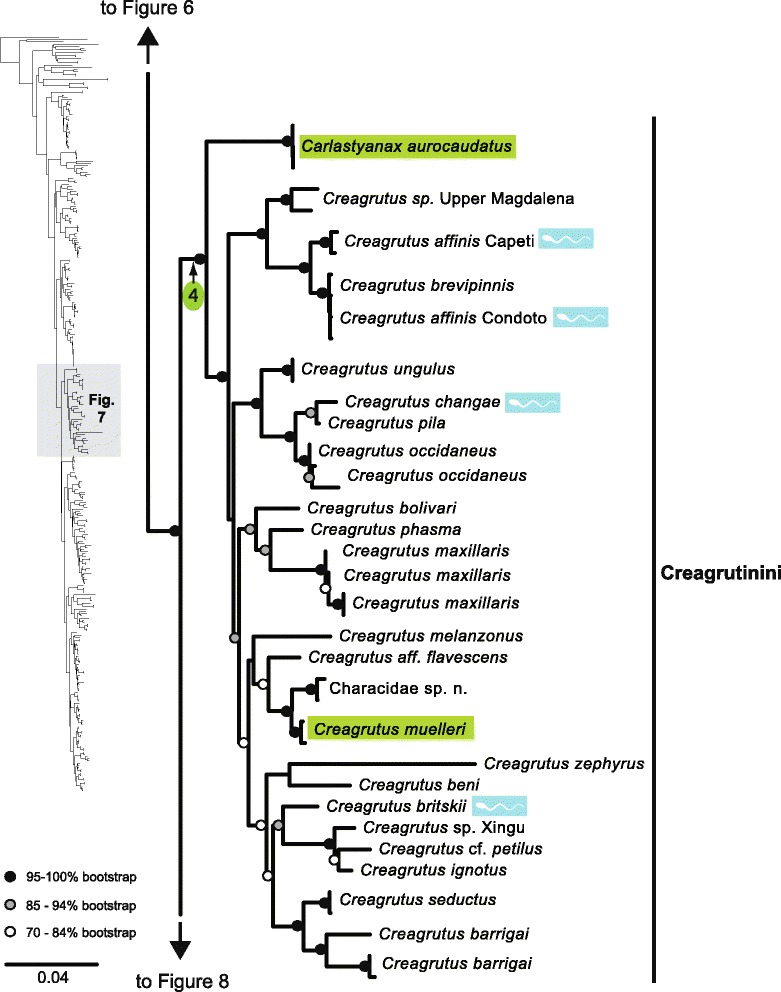
ᅟ

**Fig. 8:**
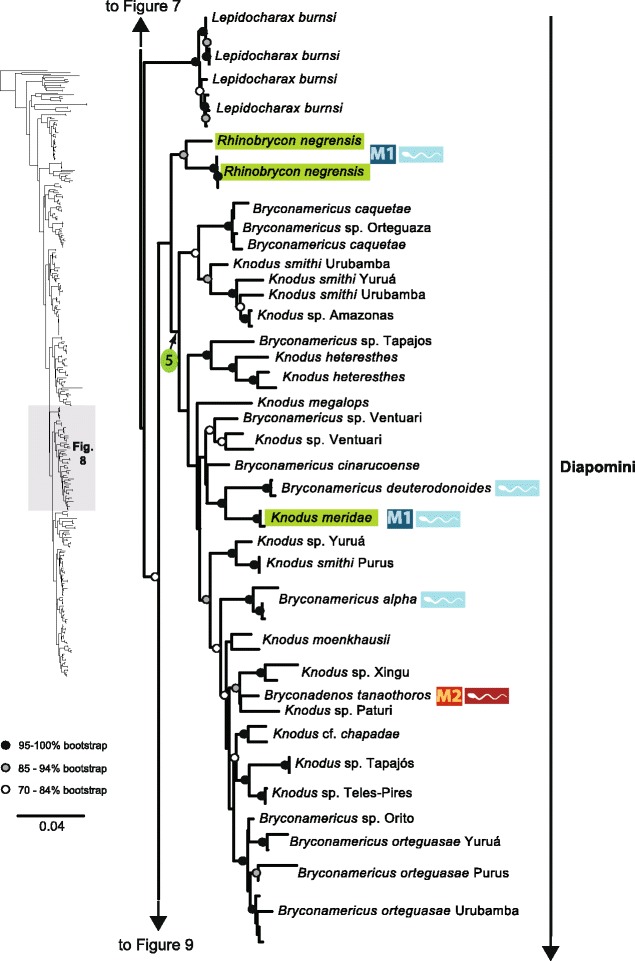
ᅟ

**Fig. 9:**
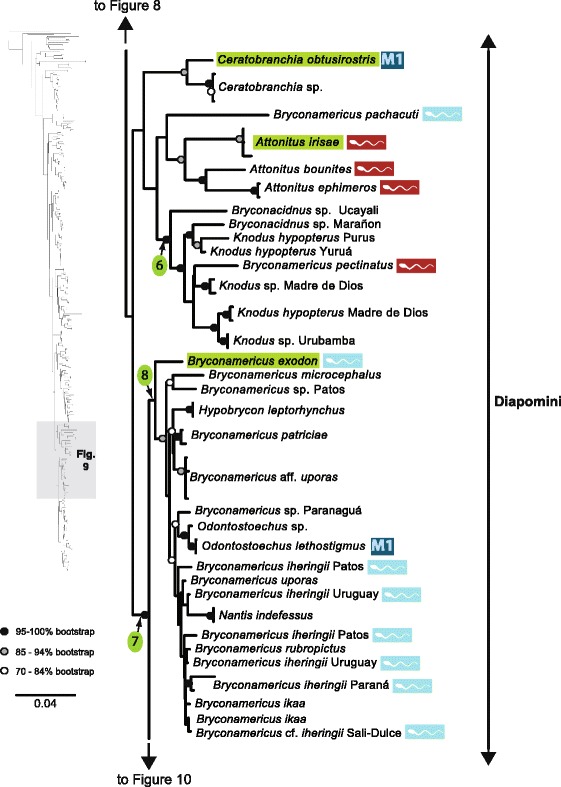
ᅟ

**Fig. 10:**
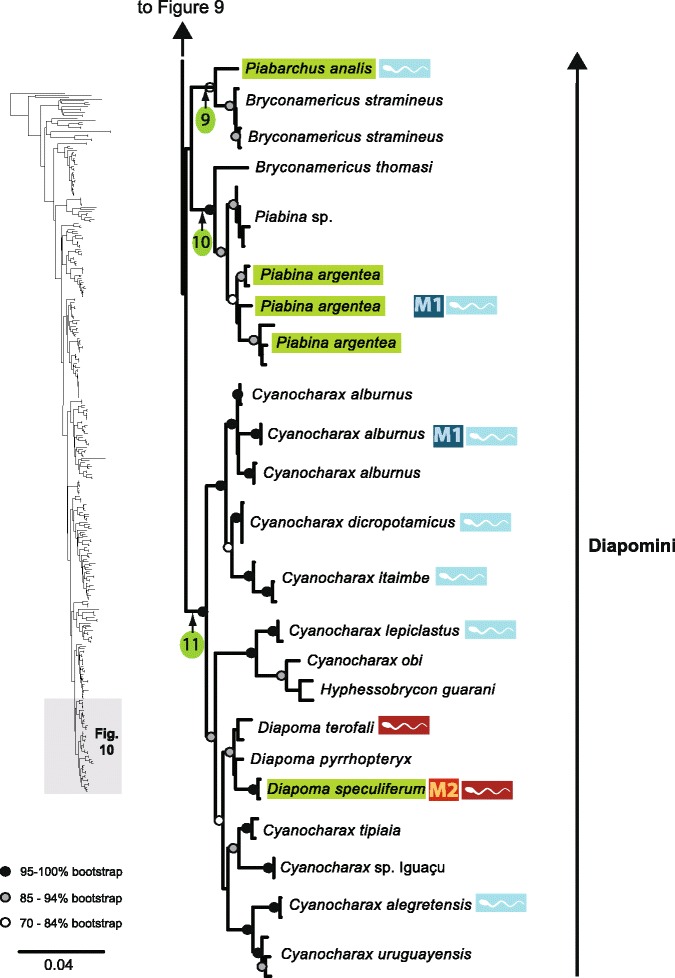
ᅟ

## Additional files

Additional file 4:
**Unabridged ML tree obtained with RAxML.** Labels correspond to specimens listed in Additional file 2. Bootstrap values (%) are placed next to internal branches. Sections of the phylogeny presented in Figures 4 to 10 are marked. (PDF 687 kb) Additional file 5:
**New Stevardiinae classification.** New classification of the subfamily Stevardiinae based on phylogenetic relationships proposed herein (Figures 4-10 is summarized in Figure 3 in the original manuscript). Taxa not examined in the current study are tentatively assigned to tribes and genera based on previous studies (see discussion for details). T: denotes the type species of the genus. [NC]: species assigned to a different genus (NEW COMBINATION). (DOCX 130 kb)
