# Supplementary material for: Molecular phylogeny of the subfamily Stevardiinae Gill, 1858 (Characiformes: Characidae): classification and the evolution of reproductive traits
Source: BMC Evol Biol. 2015 Jul 21;15:146. doi: 10.1186/s12862-015-0403-4 (PMC4509481; doi:10.1186/s12862-015-0403-4)
Supplement: Additional file 1: — Nomenclatural remarks regarding Stevardiinae, Glandulocaudinae and Corynopomini. [file 12862_2015_403_MOESM1_ESM.docx]

**Additional file 1 – Nomenclatural Remarks**

The family group name Stevardiinae was first proposed in 1858 by Gill [1] (misspelled Stevardianae) to include *Stevardia*, *Corynopoma* and *Nematopoma,* described in the same work. In the first review of these genera, *Corynopoma* was proposed as the senior synonym of *Stevardia* and *Nematopoma* [2] despite the fact that *Stevardia* has page precedence in Gill’s paper (see [3] for discussion). According to the ICZN [4] (Article 40.1), even though the type genus *Stevardia* becomes a junior synonym of *Corynopoma*, the family group name Stevardiinae is not affected and should not be replaced. In 1914, Eigenmann [5], however, created the subfamily name Glandulocaudinae for characids bearing a caudal glandular organ in males (including *Corynopoma*) and Stevardiinae, that has precedence over Glandulocaudinae, remained ignored for decades.

The ICZN [4] (article 35.5) establishes that “If after 1999 a name in use for a family-group taxon (*e.g.*, for a subfamily) is found to be older than a name in prevailing usage for a taxon at higher rank in the same family-group taxon (*e.g.,* for the family within which the older name is the name of a subfamily) the older name is not to displace the younger name”. In 2005 [6] the subfamily name Stevardiinae was resurrected as a subfamily different from the Glandulocaudinae and this act does not conflict with ICZN rules [4]. In 2010, Mirande [7] named “clade A” (that includes Stevardiinae and Glandulocaudinae *sensu* Weitzman, Menezes, Evers & Burns [6]), as Stevardiinae and in our view this does not conflict ICZN [4] (article 35.5) since the name Stevardiinae has not been treated previously as subordinated to Glandulocaudinae. Since *Corynopoma* and *Stevardia* are synonyms, the tribe Corynopomini Eigenmann, 1927 automatically turns to be a junior synonym of Stevardiini Gill, 1878.

**References**

1. Gill TN. Synopsis of the fresh water fishes of the western portion of the island of Trinidad, W. I. Annals of the Lyceum of Natural History of New York. 1858; 6: 363–430.

2. Günther A. Catalogue of the Fishes in the British Museum, Volume 5. London: Trustees of the British Museum; 1864. 455p.

3. Weitzman SH, Fink SV. Xenurobryconin phylogeny and putative pheromone pumps in glandulocaudine fishes (Teleostei, Characidae). Smithsonian Contrib Zool. 1985; 421: 1–121.

4. ICZN. International Code on Zoological Nomenclature. The International Trust for Zoological Nomenclature. London: Natural History Museum; 1999.

5. Eigenmann CH. The Glandulocaudinae (a new subfamily of characid fishes with innate potentialities for sexual dimorphism). South American Fishes. Indiana University; 1914. 32–42.

6. Weitzman SH, Menezes NA, Evers HG, Burns JR. Putative relationships among inseminating and externally fertilizing characids, with a description of a new genus and species of brazilian inseminating fish bearing an anal-fin gland in males (Characiformes: Characidae). Neotrop Ichthyol. 2005; 3: 329–60.

7. Mirande JM. Phylogeny of the family Characidae (Teleostei: Characiformes): from characters to taxonomy. Neotrop Ichthyol. 2010; 8: 385–568.
