# Supplementary figures and images for: Molecular phylogeny of the subfamily Stevardiinae Gill, 1858 (Characiformes: Characidae): classification and the evolution of reproductive traits
Source: BMC Evol Biol. 2015 Jul 21;15:146. doi: 10.1186/s12862-015-0403-4 (PMC4509481; doi:10.1186/s12862-015-0403-4)

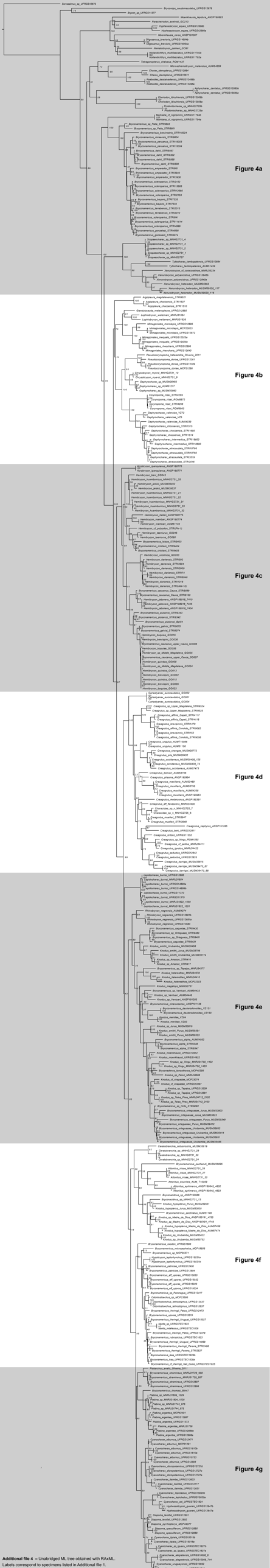

Supplement: Additional file 4: — Unabridged ML tree obtained with RAxML. [file 12862_2015_403_MOESM4_ESM.pdf]
