# Supplementary figures and images for: Erratum to: Molecular phylogeny of the subfamily Stevardiinae Gill, 1858 (Characiformes: Characidae): classification and the evolution of reproductive traits
Source: BMC Evol Biol. 2015 Dec 3;15:269. doi: 10.1186/s12862-015-0489-8 (PMC4669599; doi:10.1186/s12862-015-0489-8)

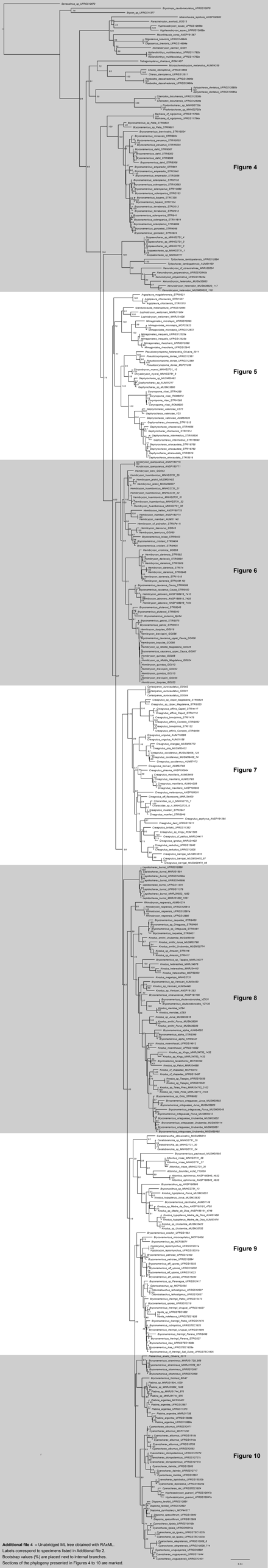

Supplement: Additional file 4: — Unabridged ML tree obtained with RAxML. Labels correspond to specimens listed in Additional file 2. Bootstrap values (%) are placed next to internal branches. Sections of the phylogeny presented in Figures 4 to 10 are marked. (PDF 687 kb) [file 12862_2015_489_MOESM4_ESM.pdf]
