# Supplementary material for: Erratum to: Molecular phylogeny of the subfamily Stevardiinae Gill, 1858 (Characiformes: Characidae): classification and the evolution of reproductive traits
Source: BMC Evol Biol. 2015 Dec 3;15:269. doi: 10.1186/s12862-015-0489-8 (PMC4669599; doi:10.1186/s12862-015-0489-8)
Supplement: Additional file 5: — New Stevardiinae classification. New classification of the subfamily Stevardiinae based on phylogenetic relationships proposed herein (Figures 4-10 is summarized in Figure 3 in the original manuscript). Taxa not examined in the current study are tentatively assigned to tribes and genera based on previous studies (see discussion for details). T: denotes the type species of the genus. [NC]: species assigned to a different genus (NEW COMBINATION). (DOCX 130 kb) [file 12862_2015_489_MOESM5_ESM.docx]

**Additional file 5 – New Stevardiinae classification**

New classification of the subfamily Stevardiinae based on phylogenetic relationships proposed herein (Figures 4-10 is summarized in Figure 3 in the original manuscript). Taxa not examined in the current study are tentatively assigned to tribes and genera based on previous studies (see discussion for details). ^T^: denotes the type species of the genus. [NC]: species assigned to a different genus (NEW COMBINATION).

**SUBFAMILY STEVARDIINAE**

**Tribe Eretmobryconini** NEW TRIBE (Figure 4) [13 species]

***Eretmobrycon*** Fink, 1976

^T^*Eretmobrycon* *bayano* Fink, 1976

*Eretmobrycon* *brevirostris* (Günther, 1860) [NC]

*Eretmobrycon* *dahli* (Román-Valencia, 2000) [NC]

*Eretmobrycon* *emperador* (Eigenmann and Ogle, 1907) [NC]

*Eretmobrycon* *gonzalezi* (Román-Valencia, 2002) [NC]

*Eretmobrycon* *miraensis* (Fowler, 1945) [NC]

*Eretmobrycon* *peruanus* (Müller and Troschel, 1845) [NC]

*Eretmobrycon* *scleroparius* (Regan, 1908) [NC]

*Eretmobrycon* *terrabensis* (Meek, 1914) [NC]

Not examined but likely included in *Eretmobrycon*: *Bryconamericus* *guaytarae* Eigenmann and Henn, 1914, *Bryconamericus* *simus* (Boulenger, 1898).

***Markiana*** Eigenmann, 1903

^T^*Markiana nigripinnis* (Perugia, 1891)

Not examined but likely included in *Markiana: Markiana geayi* (Pellegrin, 1909).

**Tribe Xenurobryconini** Myers and Böhlke, 1956 - *sensu* Weitzman and Fink, 1985 (Figure 4) [13 species]

***Scopaeocharax*** Weitzman and Fink, 1985

*Scopaeocharax* sp. from the Huallaga River basin (Perú)

Not examined but likely included in *Scopaeocharax*: *Scopaeocharax atopodus* (Böhlke, 1958), ^T^*Scopaeocharax rhinodus* (Böhlke, 1958) reported from the same basin as the examined specimens.

***Tyttocharax*** Fowler, 1913

*Tyttocharax tambopatensis* Weitzman and Ortega, 1995

Not examined but likely included in *Tyttocharax: Tyttocharax cochui* (Ladiges, 1949), ^T^*Tyttocharax madeira* Fowler, 1913, *Tyttocharax metae* Román-Valencia, García-Alzate, Ruiz-C. and Taphorn, 2012.

***Xenurobrycon*** Myers and Miranda Ribeiro, 1945

*Xenurobrycon coracoralinae* Moreira, 2005

*Xenurobrycon heterodon* Weitzman and Fink, 1985

*Xenurobrycon polyancistrus* Weitzman, 1987

Not examined but likely included in *Xenurobrycon*: ^T^*Xenurobrycon macropus* Myers and Miranda Ribeiro, 1945, *Xenurobrycon pteropus* Weitzman and Fink, 1985.

Not examined but likely included in *Xenurobryconini:* ^T^*Iotabrycon praecox* Roberts, 1973, ^T^*Ptychocharax rhyacophila* Weitzman, Fink, Machado-Allison and Royero L., 1994.

**Tribe Glandulocaudini** Eigenmann, 1914 - *sensu* Menezes and Weitzman, 2009 (Figure 5) [10 species]

***Glandulocauda*** Eigenmann, 1911

^T^*Glandulocauda melanopleura* (Ellis, 1911)

Not examined but likely included in *Glandulocauda*: *Glandulocauda caerulea* Menezes and Weitzman, 2009.

***Lophiobrycon*** Castro Ribeiro, Benine and Melo, 2003

^T^*Lophiobrycon weitzmani* Castro, Ribeiro, Benine and Melo, 2003

***Mimagoniates*** Regan, 1907

*Mimagoniates inequalis* (Eigenmann, 1911)

*Mimagoniates microlepis* (Steindachner, 1877)

*Mimagoniates rheocharis* Menezes and Weitzman, 1990

Not examined but likely included in *Mimagoniates*: ^T^*Mimagoniates barberi* Regan, 1907, *Mimagoniates lateralis* (Nichols, 1913), *Mimagoniates pulcher* Menezes and Weitzman, 2009, *Mimagoniates sylvicola* Menezes and Weitzman, 1990.

**Tribe Stevardiini** Gill, 1878 NEW CIRCUMSCRIPTION (Figure 5) [23 species]

***Chrysobrycon*** Weitzman and Menezes, 1998

*Chrysobrycon myersi* (Weitzman and Thomerson, 1970)

Not examined but likely included in *Chrysobrycon*: *Chrysobrycon eliasi* Vanegas-Ríos, Azpelicueta and Ortega, 2011, ^T^*Chrysobrycon hesperus* (Böhlke, 1958).

***Corynopoma*** Gill, 1858

^T^*Corynopoma riisei* Gill, 1858

***Gephyrocharax*** Eigenmann, 1912

*Gephyrocharax atracaudata* (Meek and Hildebrand, 1912)

^T^*Gephyrocharax chocoensis* Eigenmann, 1912

*Gephyrocharax intermedius* Meek and Hildebrand, 1916

*Gephyrocharax valencia* Eigenmann, 1920

Not examined but likely included in *Gephyrocharax: Gephyrocharax caucanus* Eigenmann, 1912*, Gephyrocharax chaparae* Fowler, 1940*, Gephyrocharax major* Myers, 1929*, Gephyrocharax martae* Dahl, 1943*, Gephyrocharax melanocheir* Eigenmann, 1912*, Gephyrocharax chaparae* Fowler, 1940*, Gephyrocharax sinuensis* Dahl, 1964*, Gephyrocharax torresi* Vanegas-Ríos, Azpelicueta, Mirande and García Gonzales, 2013*, Gephyrocharax venezuelae* Schultz, 1944*, Gephyrocharax whaleri* Hildebrand, 1938.

***Pseudocorynopoma*** Perugia, 1891

^T^*Pseudocorynopoma doriae* Perugia, 1891

*Pseudocorynopoma heterandria* Eigenmann, 1914

Not examined but likely included in Corynopomini: ^T^*Hysteronotus megalostomus* Eigenmann, 1911, ^T^*Pterobrycon landoni* Eigenmann, 1913, *Pterobrycon myrnae* Bussing, 1974.

**Tribe Hemibryconini** Géry, 1966 NEW CIRCUMSCRIPTION (Figure 6) [40 species]

***Acrobrycon*** Eigenmann and Pearson, 1924

^T^*Acrobrycon ipanquianus* (Cope, 1877)

Not examined but likely included in *Acrobrycon: Acrobrycon ortii* Arcila, Vari and Menezes, 2014*, Acrobrycon starnesi* Arcila, Vari and Menezes, 2014.

***Hemibrycon*** Günther, 1864

*Hemibrycon beni* Pearson, 1924

*Hemibrycon boquiae* (Eigenmann, 1913)

*Hemibrycon* *caucanus* (Eigenmann, 1913) [NC]

*Hemibrycon* *cristiani* (Román-Valencia, 1999) [NC]

*Hemibrycon dariensis* Meek and Hildebrand, 1916

*Hemibrycon galvisi* (Román-Valencia, 2000) [NC]

*Hemibrycon helleri* Eigenmann, 1927

*Hemibrycon huambonicus* (Steindachner, 1882)

*Hemibrycon inambari* Bertaco and Malabarba, 2010

*Hemibrycon jabonero* Schultz, 1944

*Hemibrycon jelskii* (Steindachner, 1876)

*Hemibrycon* *loisae* (Géry 1964) [NC]

*Hemibrycon* *plutarcoi* (Román-Valencia 2001) [NC]

^T^*Hemibrycon polyodon* (Günther 1864)

*Hemibrycon taeniurus* (Gill 1858)

*Hemibrycon virolinica* Román-Valencia and Arcila-Mesa 2010

Not examined but likely included in *Hemibrycon*: *Hemibrycon antioquiae* Román-Valencia, Ruiz-C., Taphorn, Mancera-Rodriguez and García-Alzate 2013, *Hemibrycon cairoense* Román-Valencia and Arcila-Mesa 2009, *Hemibrycon cardalensis* Román-Valencia, Ruiz-C., Taphorn, Mancera-Rodriguez, García-Alzate 2013, *Hemibrycon carrilloi* Dahl, 1960, *Hemibrycon colombianus* Eigenmann, 1914, *Hemibrycon decurrens* (Eigenmann, 1913), *Hemibrycon dentatus* (Eigenmann, 1913), *Hemibrycon divisorensis* Bertaco, Malabarba, Hidalgo and Ortega, 2007, *Hemibrycon fasciatus* Román-Valencia, Ruiz-C., Taphorn P., Mancera-Rodriguez and García-Alzate, 2013, *Hemibrycon metae* Myers 1930, *Hemibrycon mikrostiktos* Bertaco and Malabarba 2010, *Hemibrycon paez* Román-Valencia and Arcila-Mesa 2010, *Hemibrycon palomae* Román-Valencia, Garcia-Alzate, Ruiz-C. and Taphorn 2010, *Hemibrycon rafaelense* Román-Valencia and Arcila-Mesa 2008, *Hemibrycon raqueliae* Román-Valencia and Arcila-Mesa 2010, *Hemibrycon surinamensis* Géry 1962, *Hemibrycon tridens* Eigenmann 1922, *Hemibrycon velox* Dahl 1964, *Hemibrycon yacopiae* Román-Valencia and Arcila-Mesa 2010.

Not examined but likely included in Hemibryconini: ^T^*Boehlkea fredcochui* Géry, 1966, *Boehlkea orcesi* (Böhlke, 1958).

**Tribe Creagrutini** Miles, 1943 NEW CIRCUMSCRIPTION (Figure 7) [72 SPECIES]

***Carlastyanax*** Géry, 1972

^T^*Carlastyanax aurocaudatus* Eigenmann 1913

***Creagrutus*** Günther, 1864

*Creagrutus affinis* Steindachner 1880

*Creagrutus barrigai* Vari and Harold 2001

*Creagrutus beni* Eigenmann 1911

*Creagrutus bolivari* Schultz 1944

*Creagrutus brevipinnis* Eigenmann 1913

*Creagrutus britskii* Vari and Harold 2001

*Creagrutus changae* Vari and Harold 2001

*Creagrutus flavescens* Vari and Harold 2001

*Creagrutus ignotus* Vari and Harold 2001

*Creagrutus maxillaris* (Myers 1927)

*Creagrutus melanzonus* Eigenmann 1909

^T^*Creagrutus muelleri* (Günther 1859)

*Creagrutus occidaneus* Vari and Harold 2001

*Creagrutus petilus* Vari and Harold 2001

*Creagrutus phasma* Myers 1927

*Creagrutus pila* Vari and Harold 2001

*Creagrutus seductus* Vari and Harold 2001

*Creagrutus ungulus* Vari and Harold 2001

*Creagrutus zephyrus* Vari and Harold 2001

Not examined but likely included in *Creagrutus*: *Creagrutus amoenus* Fowler 1943, *Creagrutus anary* Fowler 1913, *Creagrutus atratus* Vari and Harold 2001, *Creagrutus atrisignum* Myers 1927, *Creagrutus calai* Vari and Harold 2001, *Creagrutus caucanus* Eigenmann 1913, *Creagrutus cochui* Géry 1964, *Creagrutus cracentis* Vari and Harold 2001, *Creagrutus crenatus* Vari and Harold 2001, *Creagrutus ephippiatus* Vari and Harold 2001, *Creagrutus figueiredoi* Vari and Harold 2001, *Creagrutus gephyrus* Böhlke and Saul 1975, *Creagrutus gracilis* Vari and Harold 2001, *Creagrutus guanes* Torres-Mejia and Vari 2005, *Creagrutus gyrospilus* Vari and Harold 2001, *Creagrutus hildebrandi* Schultz 1944, *Creagrutus holmi* Vari and Harold 2001, *Creagrutus hysginus* Harold, Vari, Machado-Allison and Provenzano 1994, *Creagrutus kunturus* Vari, Harold and Ortega 1995, *Creagrutus lassoi* Vari and Harold 2001, *Creagrutus lepidus* Vari, Harold, Lasso and Machado-Allison 1993, *Creagrutus leuciscus* Regan 1913, *Creagrutus machadoi* Vari and Harold 2001, *Creagrutus maculosus* Román-Valencia, García-Alzate, Ruiz-C. and Taphorn 2010, *Creagrutus magdalenae* Eigenmann 1913, *Creagrutus magoi* Vari and Harold 2001, *Creagrutus manu* Vari and Harold 2001, *Creagrutus maracaiboensis* (Schultz 1944), *Creagrutus melasma* Vari, Harold and Taphorn 1994, *Creagrutus menezesi* Vari and Harold 2001, *Creagrutus meridionalis* Vari and Harold 2001, *Creagrutus molinus* Vari and Harold 2001, *Creagrutus mucipu* Vari and Harold 2001, *Creagrutus nigrostigmatus* Dahl 1960, *Creagrutus nigrotaeniatus* Dagosta and Pastana 2014, *Creagrutus ortegai* Vari and Harold 2001, *Creagrutus ouranonastes* Vari and Harold 2001, *Creagrutus paraguayensis* Mahnert and Géry 1988, *Creagrutus paralacus* Harold and Vari 1994, *Creagrutus pearsoni* Mahnert and Géry 1988, *Creagrutus peruanus* (Steindachner 1876), *Creagrutus planquettei* Géry and Renno 1989, *Creagrutus provenzanoi* Vari and Harold 2001, *Creagrutus runa* Vari and Harold 2001, *Creagrutus saxatilis* Vari and Harold 2001, *Creagrutus taphorni* Vari and Harold 2001, *Creagrutus tuyuka* Vari and Lima 2003, *Creagrutus varii* Ribeiro, Benine and Figueiredo 2004, *Creagrutus veruina* Vari and Harold 2001, *Creagrutus vexillapinnus* Vari and Harold 2001, *Creagrutus xiphos* Vari and Harold 2001, *Creagrutus yanatili* Harold and Salcedo 2010.

**Tribe Diapomini** Eigenmann, 1909 NEW CIRCUMSCRIPTION (Figs. 8-10) [90 SPECIES]

***Attonitus*** Vari and Ortega, 2000 (Figure 9)

*Attonitus bounites* Vari and Ortega 2000

*Attonitus ephimeros* Vari and Ortega 2000

^T^*Attonitus irisae* Vari and Ortega 2000

***“Bryconacidnus”*** clade (node 6, Figure 9)

***Bryconacidnus*** Myers, 1929 *sedis mutabilis*

*Bryconacidnus* sp. - two unidentified specimens from the Ucayali and Marañon rivers (Perú)

*“Bryconamericus” pectinatus* (Vari and Siebert 1990)

*“Knodus” hypopterus* (Fowler 1943)

Not examined but likely included in *Bryconacidnus* clade: ^T^*Bryconacidnus ellisi* (Pearson 1924), *Bryconacidnus hemigrammus* (Pearson 1924), *Bryconacidnus paipayensis* (Pearson 1929).

***Bryconamericus*** ***sensu stricto*** clade (node 8, Figure 9)

***Bryconamericus*** Eigenmann, 1907

^T^*Bryconamericus exodon* Eigenmann 1907

*Bryconamericus iheringii* (Boulenger 1887)

*Bryconamericus ikaa* Casciotta, Almirón and Azpelicueta 2004

*Bryconamericus indefessus* (Mirande, Aguilera and Azpelicueta 2006) [NC]

*Bryconamericus leptorhynchus* (da Silva and Malabarba 1996) [NC]

*Bryconamericus lethostigmus* (Gomes 1947) [NC]

*Bryconamericus microcephalus* (Miranda Ribeiro 1908)

*Bryconamericus patriciae* da Silva 2004

*Bryconamericus rubropictus* (Berg 1901)

*Bryconamericus uporas* Casciotta, Azpelicueta and Almirón 2002

Not examined but likely included in *Bryconamericus sensu stricto*: *Bryconamericus agna* Azpelicueta and Almirón 2001, *Bryconamericus ecai* da Silva 2004, *Bryconamericus eigenmanni* (Evermann and Kendall 1906), *Bryconamericus lambari* Malabarba and Kindel 1995, *Bryconamericus mennii* Miquelarena, Protogino, Filiberto and López 2002, *Bryconamericus ornaticeps* Bizerril and Perez-Neto 1995, *Bryconamericus pyahu* Azpelicueta, Casciotta and Almirón 2003, *Bryconamericus sylvicola* Braga 1998, *Bryconamericus tenuis* Bizerril and Auraujo 1992, *Bryconamericus turiuba* Langeani, Lucena, Pedrini and Tarelho-Pereira 2005, *Bryconamericus ytu* Almirón, Azpelicueta and Casciotta 2004, *Hypobrycon maromba* Malabarba and Malabarba 1994, *Hypobrycon poi* Almirón, Casciotta, Azpelicueta and Cione 2001.

*“****Bryconamericus****” pachacuti* Eigenmann 1927 (Figure 9)

***Ceratobranchia*** Eigenmann, 1914 (Figure 9)

^T^*Ceratobranchia obtusirostris* Eigenmann 1914

Not examined but likely included in *Ceratobranchia:Ceratobranchia binghami* Eigenmann 1927, *Ceratobranchia delotaenia* Chernoff and Machado-Allison 1990, *Ceratobranchia elatior* Tortonese 1942, *Ceratobranchia joanae* Chernoff and Machado-Allison 1990.

***Diapoma*** Cope, 1894 (node 11, Figure 10)

*Diapoma alburnus* (Hensel 1870) [NC]

*Diapoma alegretensis* (Malabarba and Weitzman 2003) [NC]

*Diapoma dicropotamicus* (Malabarba and Weitzman 2003) [NC]

*Diapoma guarani* (Mahnert and Géry) 1987

*Diapoma itaimbe* (Malabarba and Weitzman) 2003 [NC]

*Diapoma lepiclastus* (Malabarba, Weitzman and Casciotta) 2003 [NC]

*Diapoma obi* (Casciotta, Almirón, Piálek and Říĉan 2012) [NC]

*Diapoma pyrrhopteryx* Menezes and Weitzman 2011

^T^*Diapoma speculiferum* Cope 1894

*Diapoma terofali* (Géry 1964)

*Diapoma tipiaia* (Malabarba and Weitzman 2003) [NC]

*Diapoma uruguayensis* (Messner 1962) [NC]

Not examined but likely included in *Diapoma*: *Diapoma thauma* (Menezes and Weitzman 2011

***Knodus*** ***sensu stricto*** clade (node 5, Figure 8)

***Knodus*** Eigenmann, 1911

*Knodus tanaothoros* (Weitzman, Menezes, Evers and Burns 2005) [NC]

*Knodus* *alpha* (Eigenmann 1914) [NC]

*Knodus* *caquetae* Fowler, 1945 [NC]

*Knodus chapadae* (Fowler 1906)

*Knodus* *cinarucoense* (Román-Valencia, Taphorn and Ruiz-C. 2008) [NC]

*Knodus* *deuterodonoides* (Eigenmann 1914) [NC]

*Knodus heteresthes* (Eigenmann 1908)

*Knodus megalops* Myers 1929

^T^*Knodus meridae* Eigenmann 1911

*Knodus moenkhausii* (Eigenmann and Kennedy 1903)

*Knodus orteguasae* (Fowler 1943) [NC]

*Knodus smithi* (Fowler 1913)

Not examined but likely included in *Knodus sensu stricto*: *Bryconadenos weitzmani* Menezes, Netto-Ferreira and Ferreira 2009*, Knodus borki* Zarske 2008, *Knodus breviceps* (Eigenmann 1908), *Knodus delta* Géry 1972, *Knodus dorsomaculatus* Ferreira and Netto-Ferreira 2010, *Knodus gamma* Géry 1972, *Knodus geryi* Lima, Britski and Machado 2004, *Knodus jacunda* (Fowler 1913), *Knodus longus* Zarske and Géry 2006, *Knodus mizquae* (Fowler 1943), *Knodus pasco* Zarske 2007, *Knodus savannensis* Géry 1961, *Knodus septentrionalis* Géry 1972, *Knodus shinahota* Ferreira and Carvajal 2007, *Knodus tiquiensis* Ferreira and Lima 2006, *Knodus victoriae* (Steindachner 1907)

***Piabarchus*** Myers, 1928 (node 9, Figure 10)

^T^*Piabarchus analis* (Eigenmann 1914)

*Piabarchus stramineus* (Eigenmann 1908) [NC]

Not examined but likely included in *Piabarchus: Piabarchus torrenticola* Mahnert and Géry 1988.

***Piabina*** Reinhardt, 1867 (node 10, Figure 10)

^T^*Piabina argentea* Reinhardt 1867

*Piabina thomasi* (Fowler 1940) [NC]

Not examined but likely included in *Piabina: Piabina anhembi* da Silva and Kaefer 2003.

***Rhinobrycon*** Myers, 1944 (Figure 8)

^T^*Rhinobrycon negrensis* Myers 1944

*Incertae sedis* in Diapomini:

***Lepidocharax*** Ferreira, Menezes and Quagio-Grassiotto 2011 (Figure 8)

*Lepidocharax burnsi* Ferreira, Menezes and Quagio-Grassiotto 2011

Not examined but likely included in *Lepidocharax:* ^T^*Lepidocharax diamantina* Ferreira, Menezes and Quagio-Grassiotto 2011.

Not examined but likely included in Diapomini: *Planaltina britskii* Menezes, Weitzman and Burns 2003, *Planaltina glandipedis* Menezes, Weitzman and Burns 2003, ^T^*Planaltina myersi* Böhlke 1954.

Genus ***incertae sedis*** examined but not placed in any tribe [1 genus, 4 species]

***Argopleura*** Eigenmann, 1913 (Figure 5)

*Argopleura chocoensis* (Eigenmann 1913)

^T^*Argopleura magdalenensis* (Eigenmann 1913)

Not examined but likely included in *Argopleura:*  *Argopleura conventus* (Eigenmann 1913), *Argopleura diquensis* (Eigenmann 1913)

Genera ***incertae sedis*** not examined (not putatively placed in any tribe) [10 genera, 12 species]

*Aulixidens* Böhlke, 1952

^T^*Aulixidens eugeniae* Böhlke 1952

*Caiapobrycon* Malabarba and Vari, 2000

^T^*Caiapobrycon tucurui* Malabarba and Vari 2000

*Cyanogaster* Mattox, Britz, Toledo-Piza and Marinho, 2013

^T^*Cyanogaster noctivaga* Mattox, Britz, Toledo-Piza and Marinho 2013

*Landonia* Eigenmann and Henn, 1914

^T^*Landonia latidens* Eigenmann and Henn, 1914

*Microgenys* Eigenmann, 1903

*Microgenys lativirgata* Pearson 1927

^T^*Microgenys minuta* Eigenmann 1913

*Microgenys weyrauchi* Fowler 1945

*Othonocheirodus* Myers, 1927

^T^*Othonocheirodus eigenmanni* Myers 1927

*Phallobrycon* Menezes, Ferreira and Netto-Ferreira, 2009

^T^*Phallobrycon adenacanthus* Menezes, Ferreira and Netto-Ferreira 2009

*Phenacobrycon* Eigenmann, 1922

^T^*Phenacobrycon henni* (Eigenmann 1914)

*Rhinopetitia* Géry, 1964

^T^*Rhinopetitia myersi* Géry 1964

*Trochilocharax* Zarske, 2010

^T^*Trochilocharax ornatus* Zarske 2010

Species ***incertae sedis*** (not examined) [37 species]

Species from Bolívia, Colombia, Guiana, Peru, Venezuela, and Northern Brazil – possibly related to *Eretmobrycon, Hemibrycon* or *Knodus*:

*Bryconamericus alfredae* Eigenmann 1927

*Bryconamericus andresoi* Román-Valencia 2003

*Bryconamericus arilepis* Román-Valencia, Vanegas-Ríos and Ruiz-C. 2008

*Bryconamericus bolivianus* Pearson 1924

*Bryconamericus bucayensis* Román-Valencia, Ruiz-C., Taphorn and García-A. 2013

*Bryconamericus carlosi* Román-Valencia 2003

*Bryconamericus charalae* Román-Valencia 2005

*Bryconamericus cismontanus* Eigenmann 1914

*Bryconamericus diaphanus* (Cope 1878)

*Bryconamericus foncensis* Román-Valencia, Vanegas-Ríos and Ruiz-C. 2009

*Bryconamericus grosvenori* Eigenmann 1927

*Bryconamericus guizae* Román-Valencia 2003

*Bryconamericus guyanensis* Zarske, Le Bail and Géry 2010

*Bryconamericus huilae* Román-Valencia 2003

*Bryconamericus hyphesson* Eigenmann 1909

*Bryconamericus icelus* Dahl 1964

*Bryconamericus ichoensis* Román-Valencia 2000

*Bryconamericus lassorum* Román-Valencia 2002

*Bryconamericus macarenae* Román-Valencia, García-Alzate, Ruiz-C. and Taphorn 2010

*Bryconamericus macrophthalmus* Román-Valencia 2003

*Bryconamericus megalepis* Fowler 1941

*Bryconamericus motatanensis* Schultz 1944

*Bryconamericus multiradiatus* Dahl 1960

*Bryconamericus novae* Eigenmann and Henn 1914

*Bryconamericus orinocoense* Román-Valencia 2003

*Bryconamericus oroensis* Román-Valencia, Ruiz-C., Taphorn P. and García-A. 2013

*Bryconamericus osgoodi* Eigenmann and Allen 1942

*Bryconamericus phoenicopterus* (Cope 1872)

*Bryconamericus singularis* Román-Valencia, Taphorn and Ruiz-C. 2008

*Bryconamericus subtilisform* Román-Valencia 2003

*Bryconamericus tolimae* Eigenmann 1913

*Bryconamericus yokiae* Román-Valencia 2003

*Bryconamericus zamorensis* Román-Valencia, Ruiz-C., Taphorn P. and García-A. 2013

*Hemibrycon microformaa* Román-Valencia and Ruiz-C. 2007

*Hemibrycon santamartae* Román-Valencia, Ruiz-C., García-Alzate and Taphorn 2010
